# Hybrid Lanthanide Metal–Organic Compounds with Flavonoids: Magneto-Optical Properties and Biological Activity Profiles

**DOI:** 10.3390/ijms26031198

**Published:** 2025-01-30

**Authors:** Sevasti Matsia, Anastasios Papadopoulos, Antonios Hatzidimitriou, Lars Schumacher, Aylin Koldemir, Rainer Pöttgen, Angeliki Panagiotopoulou, Christos T. Chasapis, Athanasios Salifoglou

**Affiliations:** 1 Laboratory of Inorganic Chemistry and Advanced Materials, School of Chemical Engineering, Aristotle University of Thessaloniki, 54124 Thessaloniki, Greece; srmatsia@cheng.auth.gr (S.M.); anastasmp@cheng.auth.gr (A.P.); 2 Laboratory of Inorganic Chemistry, Department of Chemistry, Aristotle University of Thessaloniki, 54124 Thessaloniki, Greece; hatzidim@chem.auth.gr; 3 Institut für Anorganische und Analytische Chemie, Universität Münster, Corrensstrasse 30, D-48149 Münster, Germany; l_sch010@uni-muenster.de (L.S.); a_kold02@uni-muenster.de (A.K.); pottgen@uni-muenster.de (R.P.); 4 Institute of Biosciences & Applications, National Centre of Scientific Research “Demokritos”, Aghia Paraskevi, 15310 Attiki, Greece; apanagio@bio.demokritos.gr; 5 Institute of Chemical Biology, National Hellenic Research Foundation, 11635 Athens, Greece; cchasapis@eie.gr

**Keywords:** flavonoid, bovine serum albumin, lanthanides, interactions, antibacterial efficacy

## Abstract

Lanthanides have seen rapid growth in the pharmaceutical and biomedical field, thus necessitating the development of hybrid metal–organic materials capable of exerting defined biological activities. Ternary hybrid lanthanide compounds were synthesized through reaction systems of Ln(III) (Ln = La, Nd, Eu) involving the antioxidant flavonoid chrysin (Chr) and 1,10-phenanhtroline (phen) under solvothermal conditions, thus leading to pure crystalline materials. The so-derived compounds were characterized physicochemically in the solid state through analytical (elemental analysis), spectroscopic (FT-IR, UV-visible, luminescence, ESI-MS, circular dichroism, ^151^Eu Mössbauer), magnetic susceptibility, and X-ray crystallographic techniques. The analytical and spectroscopic data corroborate the 3D structure of the mononuclear complex assemblies and are in line with theoretical calculations (Bond Valence Sum and Hirshfeld analysis), with their luminescence suggesting quenching on the flavonoid-phen electronic signature. Magnetic susceptibility data suggest potential correlations, which could be envisioned, supporting future functional sensors. At the biological level, the title compounds were investigated for their (a) ability to interact with bovine serum albumin and (b) antibacterial efficacy against Gram(−) (*E. coli*) and Gram(+) (*S. aureus*) bacteria, collectively revealing distinctly configured biological profiles and suggesting analogous applications in cellular (patho)physiologies.

## 1. Introduction

For quite some time, the utilization of metals in the diagnosis and treatment of a diverse spectrum of diseases has been a steadfast practice. The unique characteristics of metals, such as their capacity to form coordination compounds with various coordination numbers, geometries, and oxidation states, as well as their capability to bind biomolecular substrates, contribute to their potential in the field of drug discovery [1]. The emergence of cisplatin and its use as an anticancer agent in 1965 [2] paved the way for further development of chemotherapeutic metal complexes. As a result, compounds containing metal ions, such as Pt, Zn, Ru, Co, and Cu, have since been ubiquitously used in the treatment of cancer, diabetes mellitus II, etc. [3,4,5,6,7,8]. Lanthanides, a metal subgroup of f-shell elements, have been at the forefront of attention pertaining to metallodrugs due to their ability to bind DNA and their antioxidant activity [9,10]. Besides their biological activity, members of the lanthanide family exhibit luminescence and magnetic properties. In that respect, applications of luminescence quenching, demonstrated by lanthanide compounds, extend across diverse domains. Moreover, a myriad of applications for lanthanide chelates, serving as luminescent labels in bioanalysis and biotechnology, have been elucidated. All these applications encompass a range of techniques, including immunoassays, DNA hybridization assays, real-time PCR, single-nucleotide polymorphism (SNP) typing, and cell imaging [11,12,13,14,15,16]. Furthermore, magnetic anisotropy in lanthanides, stemming from their “core” f orbitals, gives rise to the development of single molecule magnets (SMM), with numerous applications extending to fields involving quantum computing and the fabrication of molecular spinotropic devices [17]. Nonetheless, there exist a limited number of cases of magneto-luminescent SMMs, in contrast to the longstanding recognition of the paramagnetic and photoluminescent characteristics inherent in lanthanides [18,19]. Moreover, the magnetic characteristics exhibited by lanthanide ions, stemming from the spin of electrons in unfilled 4f orbitals, qualify them as contrast agents. Gd(III), a lanthanide ion frequently employed as a contrast agent in magnetic resonance imaging (MRI) [20], is particularly notable for its elevated paramagnetism. Beyond gadolinium ions, other lanthanides like La(III), Eu(III), and Dy(III) have found applications as contrast agents in MRI procedures. The preference for europium ions over gadolinium arises from their isoelectronic nature. In addition, Dy(III) ions serve as negative contrast agents due to their effective transverse relativity [20,21,22]. Nd(III) complexes have also gained prominence as contrast agents in electron microscopy, owing to neodymium’s resemblance to uranium in terms of their physical and chemical properties [23].

On the other hand, flavonoids, a class of secondary metabolites, have exhibited protective effects against a variety of diseases, such as cancer, Parkinson’s, Alzheimer’s, etc., due to their anti-inflammatory, antimicrobial, antioxidant, and antitumor activity [24,25,26,27]. Flavonoids include in their molecular structure a pyran ring connected to two benzene rings and can be obtained from seeds, plants, or fruits. They play a role in regulating cell growth, attracting pollinator insects, and providing protection against both biotic and abiotic stresses [28]. Cognizant of the fact that soluble and bioavailable lanthanide ions could be formulated in the presence of naturally occurring low-molecular-mass products so as to provide biological properties and reactivity, (a) sustaining and enhancing antioxidant effects, and/or (b) counteracting prooxidant aberrations in cellular physiology, research was launched in our laboratory to design and synthesize binary–ternary complex materials of lanthanides containing flavonoids. Among the plethora of flavonoids, chrysin (Chr) was chosen for its capacity to establish a natural binding site, facilitated by its pyrene and benzene rings, conducive to metal ion chelation.

The undertaken synthetic investigation of ternary Ln(III)-chrysin (Ln = La, Nd, Eu) complexes was supported by N,N’-aromatic chelators, such as 1,10-phenanthroline (phen), thereby leading to the isolation of crystalline compounds in pure form. Driven by the desire to peruse the properties of the new hybrid materials, extensive physicochemical characterization ensued, involving analytical, spectroscopic, magnetic, and X-ray crystal structural techniques among others. In an even rarer experimental approach, a Mössbauer study was pursued in the case of the Eu(III) species. The work was further supplemented by theoretical bond valence sum (BVS) and Hirshfeld calculations to delineate the (inter)intramolecular interactions within the molecular assemblies. Based on those properties, the importance of such materials in subcellular biological interactions was subsequently looked into through (a) stability and interaction studies with bovine serum albumin (BSA) and (b) theoretical calculations, projecting mechanistically essential information of the interactive character of the synthesized species with BSA. Concurrently, assessment of their biological profile in vitro, employing both Gram(−) (*E. coli*) and Gram(+) (*S. aureus*) bacteria, has shown that the new materials exhibit enhanced antibacterial activity compared to that of the free flavonoid (Chr) and lanthanide metal salts. The collective data formulate a well-defined profile of the derived materials, thus setting the stage for their use as potential agents promoting interactions with biological targets and through them as bifunctional agents in (sub)cellular (patho)physiologies.

## 2. Results

### 2.1. Synthetic Reactivity

The synthesis of compounds **1**–**3** enhances previous work in the laboratory on lanthanide–flavonoid metal–organic compounds emerging from relevant chemical reactivity [29]. The synthetic procedure adopted herein was optimized in a concentration- and temperature-dependent manner to include lanthanide (Ln(III)) metal salts, Chr acting as a ligand, and the N,N’-aromatic chelator phen in a basic reaction environment generated by triethylamine. In that context, a mixture of water–methanol solvent systems seemed to be the better choice for the reactivity investigation and subsequent isolation of crystalline materials. Specifically, compound **1** was synthesized through the reaction of La(NO_3_)_3_·xH_2_O, Chr, and phen with a molar ratio of 1:1:1 under solvothermal conditions at 100 °C for 2 h and with triethylamine as a base. The stoichiometric rendition of the reactivity is depicted below (Reaction 1):

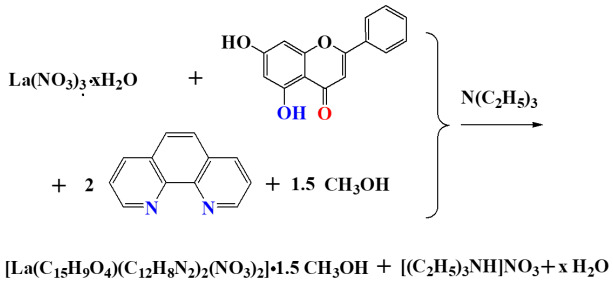
**Reaction 1**

In a similar way, the reaction of Nd(NO_3_)_3_·6H_2_O with Chr and phen, in the presence of triethylamine, led to the production of crystalline material **2**. A 1:1:1 molar ratio, higher reagent concentration, and extended reaction times under solvothermal conditions (140 °C for 48 h) were used, thus formulating the reactivity depicted below (Reaction 2).

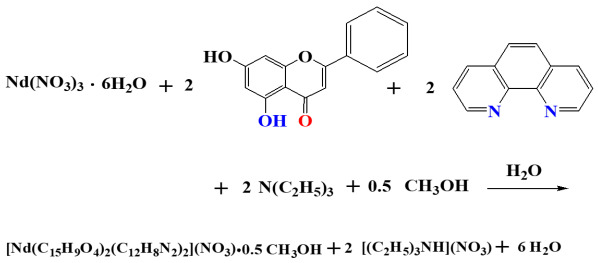
**Reaction 2**

In the case of crystalline material **3**, Eu(NO_3_)_3_·xH_2_O, Chr, and phen, in a molar ratio of 1:1:1 afforded the crystalline Eu-Chr-phen ternary compound in a basic environment created by triethylamine. The relevant stoichiometric reactivity is shown in Reaction 3.

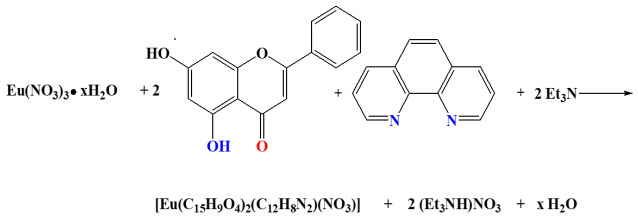
**Reaction 3**

The derived ternary Ln(III)-Chr-phen materials were easily retrieved in pure crystalline form. Elemental analysis of the isolated crystalline products projected the molecular formulation [La(C_15_H_9_O_4_)(C_12_H_8_N_2_)_2_(NO_3_)_2_]·1.5CH_3_OH (**1**), [Nd(C_15_H_9_O_4_)_2_(C_12_H_8_N_2_)_2_](NO_3_)·0.5CH_3_OH (**2**), and [Eu(C_15_H_9_O_4_)_2_(C_12_H_8_N_2_)(NO_3_)] (**3**), respectively. Further spectroscopic evaluation of the crystalline products by FT-IR confirmed the presence of ligands bound to Ln(III), thus being congruent with the proposed formulation. Finally, X-ray crystallography confirmed the analytical and spectroscopic results by unraveling the 3D structures of the crystalline products in all three cases (vide infra). Compounds **1**–**3** are slightly soluble in water and are stable in the crystalline form in air at room temperature for long periods of time.

### 2.2. Description of X-Ray Crystallographic Structures

**Figure 1:**
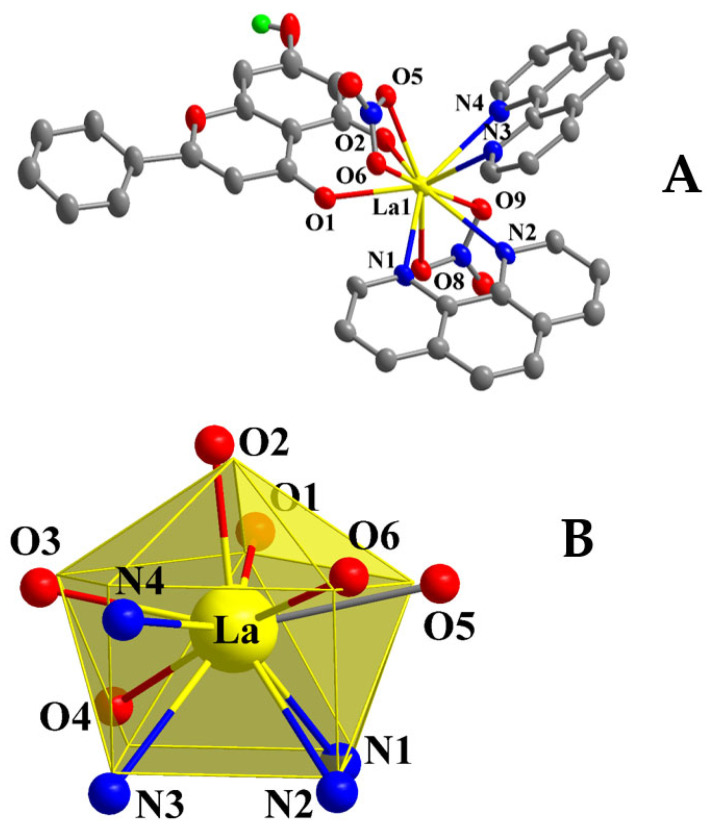
(**A**) Molecular structure of **1**; aromatic hydrogen atoms as well as solvate methanol molecules are omitted for clarity; atom colors: lanthanum, yellow; nitrogen, blue; oxygen, red; hydrogen, green. (**B**) Coordination polyhedron (real positions of the coordinated atoms together with the normal polyhedron) of **1**; atom colors: lanthanum, yellow; nitrogen, blue; oxygen, red.

Compound **1** crystallizes in the triclinic system and space group *P*ī (Table 1), with a multiplicity of 2 for the asymmetric unit. The asymmetric unit comprises one neutral mononuclear La(III) complex assembly, formulated as [La(C_15_H_9_O_4_)(C_12_H_8_N_2_)_2_(NO_3_)_2_], and one and a half solvate methanol molecules disordered over three positions with equal occupancy factors ½ each. The molecular structure is depicted in Figure 1A, and selected bond distances and angles are summarized in Table 2. Further hydrogen-bond data are shown in Table S1. The metal-bound Chr ligand is singly deprotonated and coordinated to La(III) in a bidentate chelate mode through the deprotonated phenolato (O(2)) and carbonylato (O(1)) oxygen atoms at asymmetric distances La-O of 2.376(3) and 2.442(3) Å, respectively. The two nitrogenous aromatic chelators (phen) are coordinated as usual in a bidentate chelating mode through their nitrogen atoms, with bond distances La-N in the range from 2.711(4) to 2.760(4) Å.

The coordination sphere around La(III) is completed with four additional oxygen atoms coming from two nitrato anionic ligands coordinated in a bidentate fashion, with bond distances La-O ranging from 2.613(4) to 2.706(3) Å. The La(III) ion (theoretical oxidation state calculated at 2.938 through bond valence sum calculations) is consequently ten coordinates and the complex coordination environment is thus formulated as LaO_6_N_4_. The study of the local coordination geometry with the SHAPE software (version 2) [30] revealed that the geometry around the lanthanide center adopts a distorted sphenocorona type shape, with a local symmetry C_2v_ as shown in Figure 1B. The figure presents the normal sphenocorona polyhedron with yellow edges and the real positions of the coordinated atoms. The so-emerging polyhedron comprises one tetragonal (quadrilateral after distortion) base, a second pentagonal intermediate base parallel to the first one, and a cap over the pentagonal base.

The capped top of the polyhedron is occupied by the phenolato oxygen atom, whereas the carbonyl oxygen occupies one of the pentagonal apices. The nitrogen atoms of one phen (N1 and N2) and the oxygen atoms of one nitrato ligand (O(8) and O(9)) occupy consecutive apices of the quadrilateral and the pentagonal bases, respectively. The remaining two nitrogen and two oxygen atoms occupy contiguous apices in both parallel bases.

The structure is stabilized via formation of hydrogen bonds, as the alcoholic hydrogen atoms from the solvate methanol molecules interact with the remaining alcoholic and/or nitrato oxygen atoms and the protonated phenolic group of the Chr ligand.

**Figure 2:**
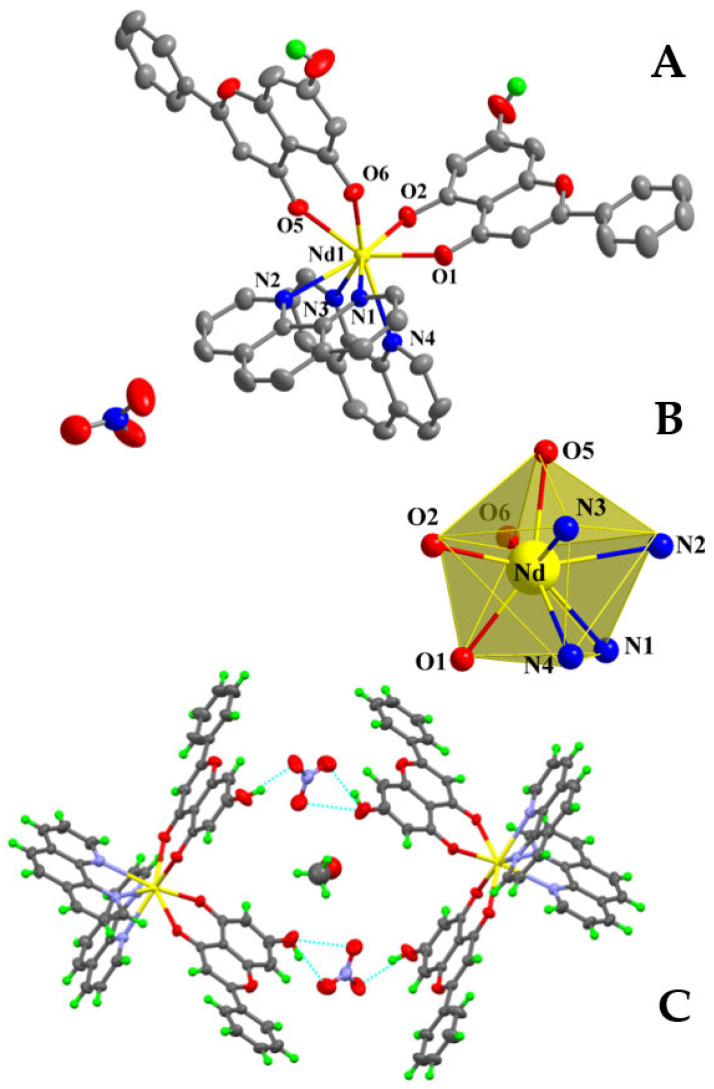
(**A**) Molecular structure of **2**; Aromatic hydrogen atoms as well as solvate methanol molecules are omitted for clarity; atom colors: neodymium, yellow; nitrogen, blue; oxygen, red; hydrogen, green. (**B**) Coordination polyhedron (real positions of the coordinated atoms together with the normal polyhedron) of **2**; atom colors: neodymium, yellow; nitrogen, blue; oxygen, red. (**C**) Hydrogen bonding interactions (blue dotted lines) in **2**.

In the case of compound **2**, crystallization in the triclinic crystal system and space group *P*ī (Table 1) provides a multiplicity of 2 for the asymmetric unit. The asymmetric unit contains one mononuclear Nd(III) singly cationic complex assembly, denoted as [Nd(C_15_H_9_O_4_)_2_(C_12_H_8_N_2_)_2_]^+^, one lattice nitrate counter ion, and a total of one-half solvate methanol molecule. The latter moiety is disordered over two positions with occupancy factors of ¼ each. The molecular structure is depicted in Figure 2A, and selected bond distances and angles are summarized in Table 2.

Both singly deprotonated Chr anionic ligands are coordinated in a bidentate chelate mode from the carbonyl and phenolato oxygen anchor atoms. The Nd-O distances are unequal, with values of (a) 2.431(3) and 2.423(3) Å for the carbonyl (longer distances), and (b) 2.304(3) and 2.291(3) Å for the phenolato (shorter distances) oxygen atoms. For the two nitrogenous aromatic chelators (phen), the coordination bond distances Nd-N are nearly equal, varying from 2.674(4) to 2.692(4) Å. Both of the phen ligands are coordinated to Nd(III) as anticipated, in a bidentate chelate mode. The coordination number around Nd(III) is eight, thus giving rise to a NdO_4_N_4_ chromophore. The study of the coordination geometry using SHAPE software revealed a distorted triangular dodecahedral geometry around the metal ion. Figure 2B presents the polyhedron of the normal triangular dodecahedron, with yellow edges and the real positions of the coordinated atoms. The best local symmetry around the Nd(III) center can be assigned as D_2d_. The oxidation state of the metal center stands at 2.718, as derived through bond valence sum calculations. Hydrogen bonding interactions (Table S1) form as a result of the protonated phenolic groups of the Chr ligands, interacting with the oxygen atoms of the nitrate anions. Consequently, closed dimers form, containing two asymmetric units and further stabilizing the structure, as shown in Figure 2C.

**Figure 3:**
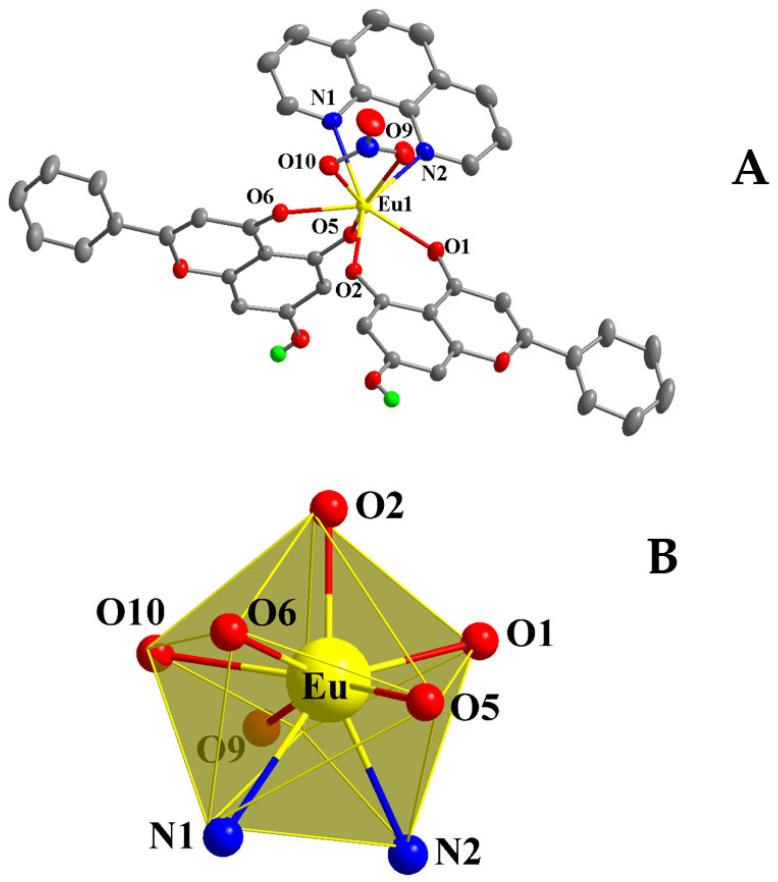
(**A**) Molecular structure of **3**; aromatic hydrogen atoms are omitted for clarity; atom colors: europium, yellow; nitrogen, blue; oxygen, red; hydrogen, green. (**B**) Coordination polyhedron (real positions of the coordinated atoms together with the normal polyhedron) of **3**; atom colors: europium, yellow; nitrogen, blue; oxygen, red.

For compound **3**, crystallization occurs in the monoclinic crystal system and space group *P*2_1_/*n* (Table 1), exhibiting a multiplicity of 4 for the asymmetric unit. The latter contains one mononuclear Eu(III) neutral complex assembly, exemplified through the formulation [Eu(C_15_H_9_O_4_)_2_(C_12_H_8_N_2_)(NO_3_)]. The molecular structure is depicted in Figure 3A. Selected bond distances and angles are summarized in Table 2.

Both Chr ligands are singly deprotonated and bound to Eu(III) in a bidentate chelate fashion as seen in the previous lanthanide complex assemblies. The phenolato oxygen atoms lie closer to the metal ion than the carbonylato oxygen. The nitrato anion is coordinated to the metal center in a bidentate chelate mode and the oxygen atoms form longer bonds to Eu(III) than the Chr bound oxygen anchors. The phen ligand is again coordinated through both nitrogen atoms in a bidentate chelate manner. The nitrogen atoms of phen exhibit the longest bond distances from Eu(III), as Eu-O bond distances vary from 2.295(2) to 2.529(3) Å and the Eu-N bond distances range from 2.596(3) to 2.604(3) Å. The Eu(III) ion is surrounded by eight coordinated atoms, with the emerging coordination sphere projecting a EuO_6_N_2_ chromophore. The europium oxidation state was found to be 2.972 through bond valence sum calculations.

The structural data on compounds **1**–**3** exhibit a well-defined range of bond distances (2.291(3)–2.760(4) Å) and angles (48.55(10)–171.43(10)°), which are in the range of those previously encountered (distances (2.263(2)–2.622(3) Å). Angles (50.13(8)–155.02(8)°)) in Ln(III)-flavonoid complexes characterized crystallographically [29].

The study of the coordination geometry using SHAPE software led to the formulation of a distorted triangular dodecahedral geometry around Eu(III), with a local symmetry of D_2d_, as shown in Figure 3B. Hydrogen bonding interactions (Table S1) emerge from the protonated phenolic groups of the Chr ligands, interacting with the coordinated phenolato oxygen atoms from neighboring complexes, thus forming an extended 3D crystal network and providing further stability to the structure.

### 2.3. Hirshfeld Surface Analysis

**Figure 4:**
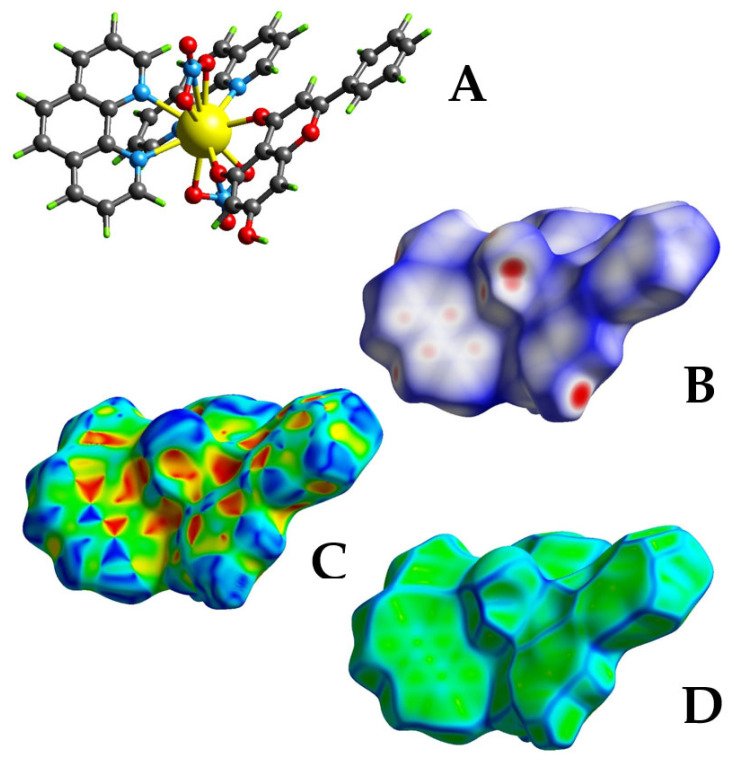
(**A**) Crystal Explorer plot of **1**. (**B**) d_norm_ mapping of **1** through Hirshfeld surface analysis. (**C**) Shape index mapping of **1** through Hirshfeld surface analysis. (**D**) Curvedness mapping of **1** through Hirshfeld surface analysis. The different colors shown in the figure are identified and explained in detail in the text.

Investigation of intermolecular and intramolecular interactions was launched for compounds **1**–**3** through Hirshfeld surface analysis, using (a) surface mapping over d_norm_, shape index, and curvedness, and (b) 2D fingerprint plots as quantitative analysis for the determination of the proportion of every type of interaction. In the case of compound **1** (Figure 4A), the ternary La-Chr-phen system shows a white-blue mapping over d_norm_ (Figure 4B) with deep red spots on the nitrato anionic ligand and phenolic group of Chr. Deep red spots show intense close O–H···O hydrogen bonds between the bound nitrato ligand and (a) the lattice methanolic moiety, and/or (b) the phenolic group of the flavonoid A neighboring ring. There are also white-blue regions shown all around the Chr and phen ligands, verifying contacts close to van der Waals radii or longer ones. Moreover, a three-color d_norm_ mapping was derived in the case of compounds **2**–**3** (Figure S2). Deep red spots are visualized in the case of **2** over the flavonoid phenolic group, thus verifying O–H···O interactions (shorter interactions) with non-coordinated lattice nitrate ligands, whereas in the mapping area of **3**, the same interactions are shown as light red spots, confirming weaker interactions between the phenolic group of Chr A ring and the external carbonyl group of abutting molecules.

Mapping over shape index (Figure 4C) for **1** and **2**–**3** (Figure S2) shows the “bow-tie” pattern of blue-red triangles, characteristic patterns of stacking arrangements, highlighting that the two molecules are in close contact. Large red regions of concave curvatures above the Chr ligand attest to characteristic patterns of C–H···π interactions, whereas the opposite curvature (convex regions of blue color) all around the organic ligands (Chr and phen) relate to C–H donor interactions.

The shape of the overall mapping surfaces (curvedness) of **1**–**3** was also investigated as shown in Figure 4D and Figure S2. There are indications of planar stacking interactions between phen moieties through the presence of big flat green surface regions as well as contacting patches highlighted as blue outlines. Moreover, curvedness surfaces of **2**–**3** are also characterized by yellow color highlights in the bound Chr, phen, and nitrato anionic ligand, corresponding to hydrogen bonds with the neighboring molecules.

**Figure 5:**
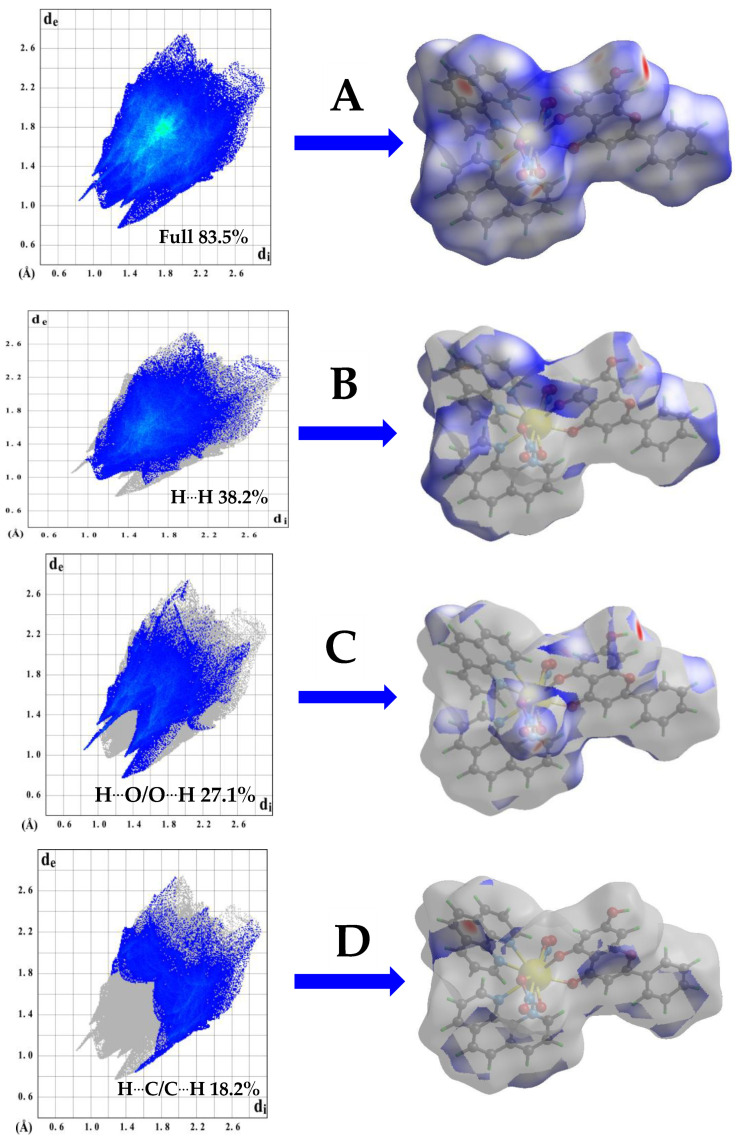
(**A**) Full fingerprint plot of **1** and d_norm_ mapping. (**B**) Internal vs. external 2D fingerprint plot distances of H···H contacts of **1** with the relevant percentage contribution mapped over d_norm_. (**C**) 2D fingerprint plot of H···O/O···H contacts and their appropriate percentage contribution reflected onto the Hirshfeld surface area mapper over d_norm_ of **1**. (**D**) 2D Fingerprint plot of H···C/C···H contacts, with the relevant percentage contribution reflected onto the Hirshfeld surface area mapper over d_norm_ of **1**.

All the aforementioned interactions have been further determined and quantitatively analyzed through full 2D fingerprint plots and their appropriate deconvolutions for **1**–**3** (Figure 5A–D and Figure S3). Specifically, in all three cases, a dense diffuse blue point plot is shown, corresponding to the highest contribution of the overall types of interactions (H···H interactions) and calculated at 38.2% for **1**, 37.6 for **2**, and 30.4 for **3**. Sharp pairs of spikes in the case of H···O interactions are calculated at 27.1% for **1**, 19.7% for **2**, and 26.3% for **3**, corresponding mostly to the interaction of the nitrate anionic ligand or phenolic group of Chr. Typical C–H···C interactions are shown as two wings in a proportion of 18.2% for **1**, 26.1% for **2**, and 27.3% for **3** of the total fingerprint plot area.

### 2.4. FT-IR Spectroscopy

FT-IR spectra for compounds **1**–**3** were recorded in the solid state, using KBr pellets, in the range of 4000 to 400 cm^−1^. A comparison between the patterns of the free ligands (Chr, phen) and the complex materials was made to confirm their complicity in each case. The primary evidence of Chr incorporation in the complex materials is the shift of the flavonoid carbonyl group (C=O), which typically appears in the nascent ligand spectra at 1653 cm^−1^, to lower frequencies. Spikes at 1635 cm^−1^, 1635 cm^−1^, and 1630 cm^−1^ for compounds **1**, **2**, and **3**, respectively, attest to the existence of the molecule in the coordination sphere of the lanthanide center. These results are in compliance with similar materials containing metal ions bound to flavonoids [29,31]. In addition, differentiation of the resonances attributed to the O-H bonds are indications of deprotonation during the chelation of the flavonoid to the lanthanide ion centers. Specifically, two broad peaks appear at 3432 and 3065 cm^−1^ for **1**, 3392 and 3058 cm^−1^ for **2**, and 3412 and 3070 cm^−1^ for **3**. Likewise, a shift of the bands attributed to the phen ligand can be observed. Sharp peaks at 1429 and 1511 cm^−1^, attributed to the phen chelator ligand, emerge at frequencies 1576 and 1533 cm^−1^ for **1**, 1580 and 1541 cm^−1^ for **2**, and 1577 and 1541 cm^−1^ for **3**. A similar shift also takes place in the range between 1338 and 1089 cm^−1^ [32]. On the contrary, in the region between the 1036 and 696 cm^−1^, there seems to be a shift to lower frequencies, accompanied by a splitting of the two bands at 843 and 735 cm^−1^ for **1**, 838 and 726 cm^−1^ for **2**, and 840 and 728 cm^−1^ for **3**, attributed to the C-H out-of-plane deformations [33]. With regard to the Ln(III) ion centers bound to organic substrates, distinct peaks of low frequencies ranging from 550 to 460 cm^−1^, in each spectrum, verify the association of the metal ion with both flavonoid and the N,N’-aromatic chelator. All of the aforementioned observations from FT-IR were consistent with X-ray crystallography.

### 2.5. UV-Visible Spectroscopy

**Figure 6:**
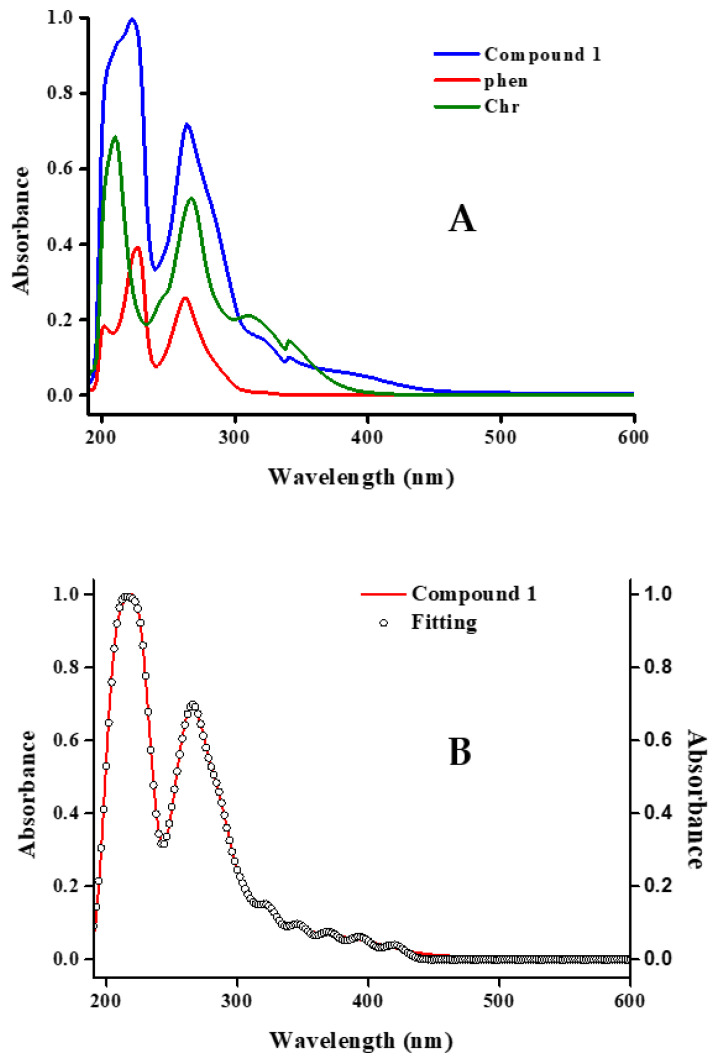
(**A**) Comparative UV-visible spectra of **1** with phen and Chr in methanol at 10^−5^ M. (**B**) Electronic spectrum (red line) and spectral fitting (scatter) of compound **1** in methanol (10^−5^ M).

Electronic spectra were recorded (190–600 nm) in (a) methanol (Figure 6A) for **1**, and (b) DMSO (Figures S4A–S6A) for compounds **1**–**3**. Further processing of electronic spectra was pursued using the Savitzky–Golay algorithm and their fitting patterns were produced. They are shown in Figure 6B and Figures S4B–S6B for methanol and DMSO, respectively. In the case of compound **1** in methanol, the spectrum (Figure 6A) shows two basic absorptions, corresponding to the absorption features of the A and B ring of Chr for π-π*, n-π*, and n-σ* transitions [34,35,36,37], the first one at 223 nm (ε 106,226 M^−1^·cm^−1^) and the second one at 265 nm (ε 73,841·M^−1^·cm^−1^), compared to that of free Chr which exhibits absorption peaks at 210 nm and 267 nm, respectively.

Those features at lower energies, from 210 to 270 nm, also cover the chelation activity of phen toward the lanthanide centers. At even higher energies, a shoulder is observed at 381 nm (ε 6934 M^−1^·cm^−1^), which is characteristic of coordinated metal centers to C=O and the phenolic group on C5 of the A-ring in Chr. In the case of DMSO, the spectra (Figures S4A–S6A) show that the characteristic peaks at lower energies disappear in both spectra (metal compounds and free organic ligands). As a result, a basic peak at 266 nm (ε 57,647 M^−1^·cm^−1^) for **1**, 269 nm (ε 55,718 M^−1^·cm^−1^) for **2**, and 267 nm (ε 49,673 M^−1^·cm^−1^) for **3** is the only one emerging in the overall pattern. A shoulder is also observed in all three patterns **1**–**3** at ~318 nm (ε N/A), related to transitions of Chr and phen ligands.

### 2.6. ESI-MS Spectrometry

**Figure 7:**
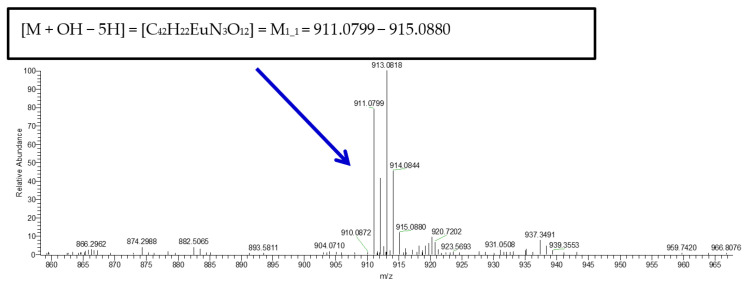
ESI-MS spectra of **3** and the appropriate species in methanol solution through the positive mode of ionization.

The ESI-MS spectra of materials **1** and **3** were recorded in the positive ionization mode, with the derived structural patterns in methanolic solution shown in Figure S7 and Figure 7, respectively. Specifically, the spectrum of **1** exhibits the following species: M1_1 = [M−phen + 2CH_3_OH + H], *m*/*z* = 825.0741–827.0792 (z = 1); M1_2 = [M − NO_3_], *m*/*z* = 814.0825–816.0878 (z = 1); M1_3 = [M − phen + CH_3_OH + OH − 3H], *m*/*z* = 742.1038–744.1091 (z = 1); M1_4 = [M − phen − CH_3_OH – OH − 2H], *m*/*z* = 645.0049–646.0078, M1_5 = [M − 2NO_3_ − phen + 2CH_3_OH − 2H], *m*/*z* = 634.0121–636.0681 (z = 1); M1_6 = [M − NO_3_ – phen − OH], *m*/*z* = 617.0223–619.0282 (z = 1). The spectrum of **3** reveals a single species, as shown in Figure 7, M3_1 = [M + OH − 5H], *m*/*z* = 911.0799–915.0880 (z = 1). The spectrum of compound **2** was not recorded through ESI-MS due to low solubility in methanol.

### 2.7. Luminescence Studies

**Figure 8:**
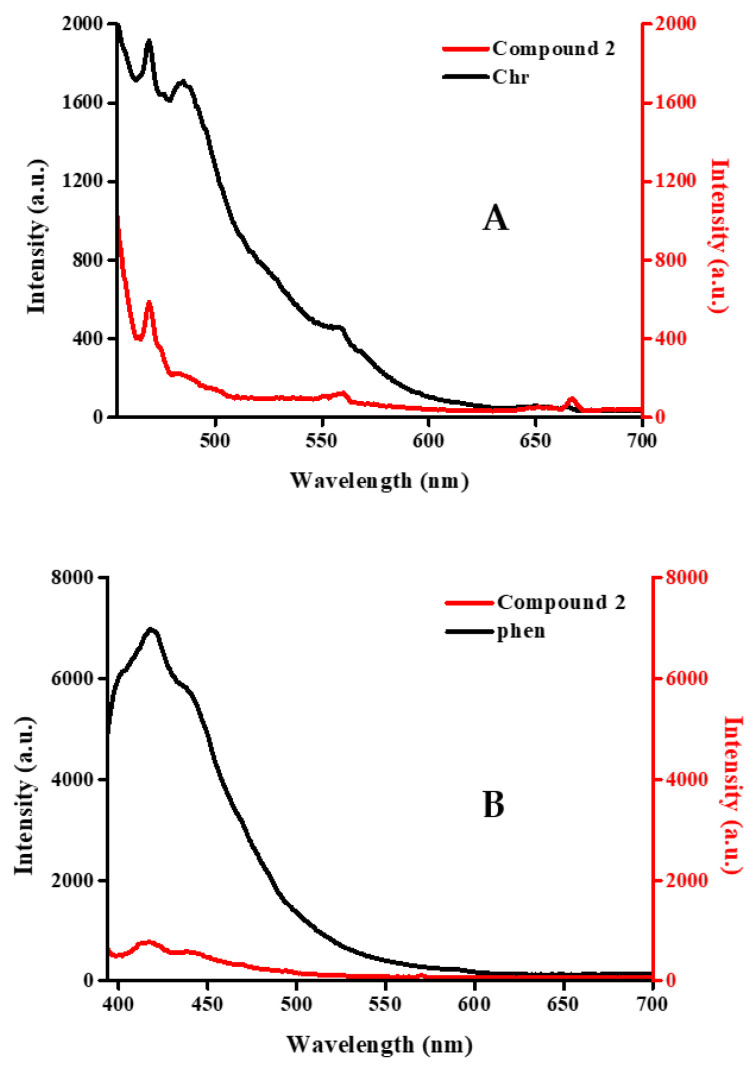
Comparative and normalized solid-state luminescence spectra between **2** and (**A**) Chr at λ_ex_ 445 nm. (**B**) Phen at λ_ex_ 373 nm.

The luminescence properties of compounds **1**–**3** were probed into in the solid state at room temperature. The excitation spectrum (Figure S8A,B) of compound **1** shows lower luminescence activity (quenching) compared to that of free Chr and phen. In the case of compounds **2** (Figure 8A,B) and **3** (Figure S9A,B), the same quenching effect was observed after investigation of the luminescence effect in comparison to the flavonoid and phen chelators. Specifically, with regard to Chr, a quenching effect emerged in all cases at λ_em_ 469 nm (compound **1** λ_ex_ 443, compound **2** λ_ex_ 445, and compound **3** λ_ex_ 385 nm) [38]. The characteristic absorption related to phen was observed as a lower intensity peak at λ_em_ 417 nm with compound **1** λ_ex_ 379 nm, compound **2** λ_ex_ 373, and compound **3** λ_ex_ 364 nm.

### 2.8. Magnetic Properties

**Figure 9:**
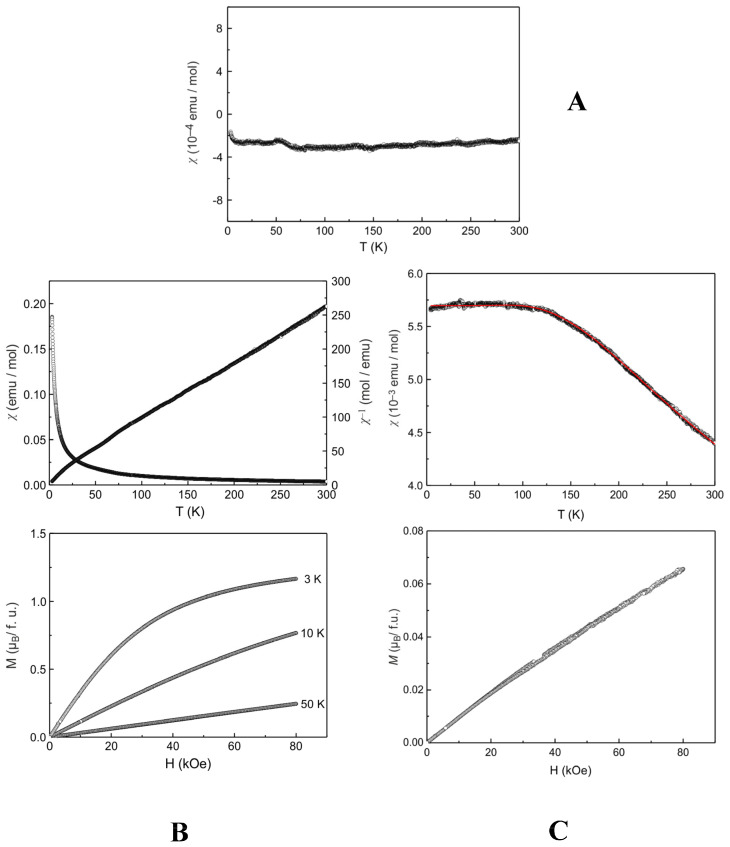
(**A**) Temperature dependence of the magnetic susceptibility of **1** measured at 10 kOe. (**B**) Magnetic properties of **2**: (**top**) temperature dependence of the magnetic susceptibility (*χ* and *χ*^−1^ data) measured at 10 kOe; (**bottom**) magnetization isotherms at 3, 10, and 50 K. (**C**) Magnetic properties of **3**: (**top**) temperature dependence of the magnetic susceptibility measured at 10 kOe. The calculated susceptibilities (red line) were obtained using the Van Vleck expression for the paramagnetic susceptibilities of free Eu(III) ions with λ = 734(1) K; (**bottom**) magnetization isotherms at 3, 10 and 50 K.

The temperature-dependent behavior of the magnetic susceptibility (10 kOe data) of **1** is presented in Figure 9A. The susceptibilities are weak and negative over the entire temperature range, compatible with diamagnetism. The room temperature value is −2.4(1) × 10^−4^ emu·mol^−1^. The tiny upturn below 20 K (a so-called Curie tail) is due to a trace amount of paramagnetic impurities. In the case of **2**, the observed behavior is shown to be paramagnetic.

The temperature dependence of the reciprocal magnetic susceptibility (Figure 9B) follows a Curie–Weiss law. The experimental magnetic moment of µ_eff_ 3.38(1) μ_B_ is slightly reduced when compared with the free ion value of Nd(III) of 3.62 μ_B_ [39]. The Weiss constant of θ −37.5(1) K points to antiferromagnetic interactions in the paramagnetic regime. The susceptibility data gave no hint for long-range magnetic ordering. This is also consistent with the magnetization data. Magnetization isotherms were recorded at 3, 10, and 50 K (Figure 9B, bottom). At 3 K and 80 kOe the saturation magnetization of µ_sat_ 1.17(1) μ_B_ is much lower than the theoretical saturation magnetization of 3.27 μ_B_ according to g·J [39].

The susceptibility values (10 kOe data) of **3** (Figure 9C) are weak and compatible with the typical Van Vleck paramagnetic behavior of Eu(III). Trivalent europium, Eu(III) (4f^6^, S = 3, L = 3), shows seven states for the ^7^F_J_ multiplet. The ground state is ^7^F_0_. Only the lowest state is taken into account when the separation of the multiplet components is sufficiently large compared to k_B_T. On the other hand, contributions of the higher multiplet components have to be considered for higher temperatures. The paramagnetic susceptibility for Eu(III) can be expressed with the help of the Van Vleck theory [40] using the equation derived by Nagata and coworkers [41] for Eu(III):

$$\chi_{para\;}\left(free\;{Eu}^{3+}\right)=\frac{N\mu_{B}^{2}}{Z}\left(\frac{A}{3\lambda }\right)$$

where A and Z contain the contributions of the respective ground states, with more detailed information on the underlying formulae provided in the original work [41]. The coupling constant λ is the only unknown parameter and can be determined by fitting the experimental susceptibilities through the stated equation. In general, a higher value of the coupling constant λ is paralleled by less hybridization between the excited state and the non-magnetic ground state.

The fit (red line in Figure 9C, top) of the experimental susceptibility data of **3** in the range 3–300 K, using the equation given above, yielded a coupling constant of λ 734(1) K. This value is in good agreement with other Eu(III) containing solids or coordination compounds, e.g., EuF_3_ (λ = 490 K) [41], EuBO_3_ (λ = 471 K) [41], Eu_2_Cl_5_(OH)(H_2_O)(terpy) (terpy = 2,2′:6,2″-terpyridine) [42] or pyrochlore-type Eu_2_Ta_2_O_7.1_N_0.6_ [43].

The room temperature susceptibility per europium ion of **3** is 4.4(1) × 10^−3^ emu·mol^−1^. This corresponds to an experimental moment of 3.20(1) μ_B_. The trivalent europium ground state is also manifested by a tiny magnetization value of 0.066(1) μ_B_·Eu·atom^−1^ at 4 K and 80 kOe (Figure 9C, bottom). 

### 2.9. ^151^Eu Mössbauer Spectroscopy

**Figure 10:**
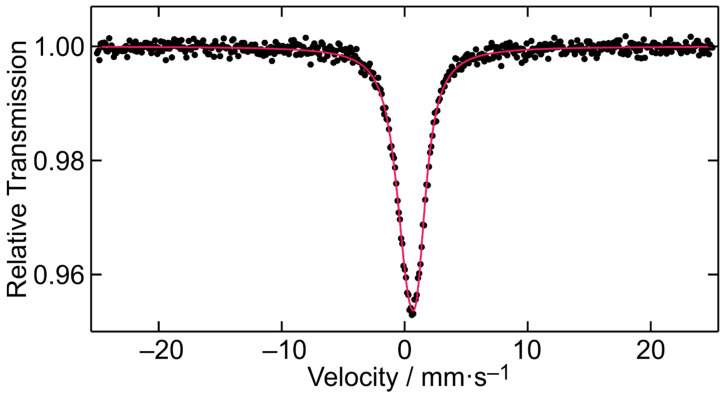
Experimental (data points) and simulated (red line) ^151^Eu Mössbauer spectrum of **3** measured at 78 K.

The ^151^Eu Mössbauer spectrum of **3** is presented in Figure 10. The sample shows a single signal at an isomer shift δ of 0.53(1) mm·s^−1^, which clearly indicates Eu(III). Due to the coordination of each Eu(III) ion by two N and six O atoms with a non-cubic symmetry, the signal is subjected to a weak quadrupole splitting Δ*E*_Q_ of 2.34(4) mm·s^−1^. The experimental line width *Γ* of 1.92(2) mm·s^−1^ is in the typical range.

The isomer shift value matches Mössbauer spectroscopic data observed for other Eu(III) complexes with N- and O-donor ligands. Examples from the literature include Eu(phen)_2_(benzoate)_3_, Eu(phen)_2_(p-hydroxybenzoate)_3_ or Eu_2_PQQ_2_ showing isomer shifts of 0.29(5), 0.58(5), and 0.32(2) mm·s^−1^, respectively [44,45].

### 2.10. Interaction with Bovine Serum Albumin

#### 2.10.1. Electronic UV-Visible Configuration

**Figure 11:**
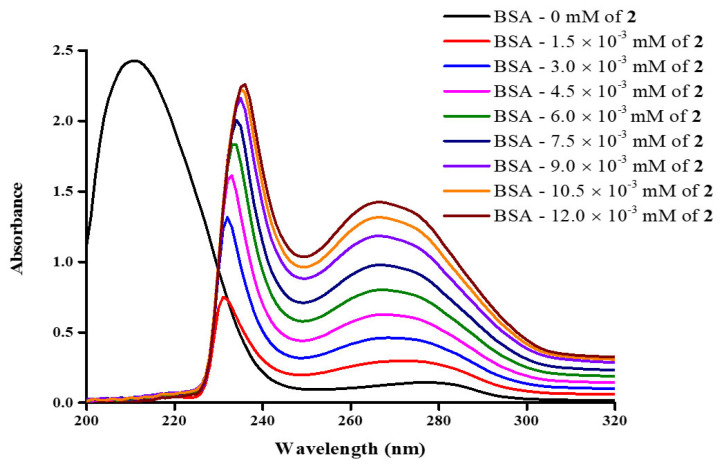
UV-visible absorption spectra of solutions containing BSA (3 μΜ, PBS) and increasing molar ratios of **2** (DMSO).

The UV-visible absorption spectra of BSA exhibit two distinct peaks at 210 and 280 nm, due to the n-π* transition of C=O in the polypeptide backbone structure and the π-π* transitions of the phenyl rings of the aromatic amino acid residues belonging to tryptophan (Trp), tyrosine (Tyr) and phenylalanine (phe), respectively [46,47,48]. Changes in the first band can be observed due to alterations of the protein secondary structure, such as the variation of the α-helical content. As reported previously, a major decrease in absorbance can be observed upon addition of metal-containing complex compounds to the BSA–buffer solution [49]. In that respect, a decrease of 70% is observed in Figure 11 for **2**, a decrease of 45% is observed for **1**, and a 52% decrease is observed for the band in **3** (Figures S10 and S11). In addition to the decrease in the 210 nm band, a major red shift is observed to 230 nm (Figure 11), with a red shift also observed to 228 nm and 225 for **1** and **3**, respectively. On the other hand, the 280 nm band is sensitive to changes in the environment around the aromatic residues. As shown in Figure 11, an increase in the absorbance of the band at 280 nm, corresponding to the increasing concentration of the lanthanide compound, could be attributed to the change of the environment surrounding the aromatic residues inside the hydrophobic pocket, thereby resulting in their exposure to the aqueous environment. These results are confirmed through CD spectra measurements (vide infra).

#### 2.10.2. Luminescence Interactions

**Figure 12:**
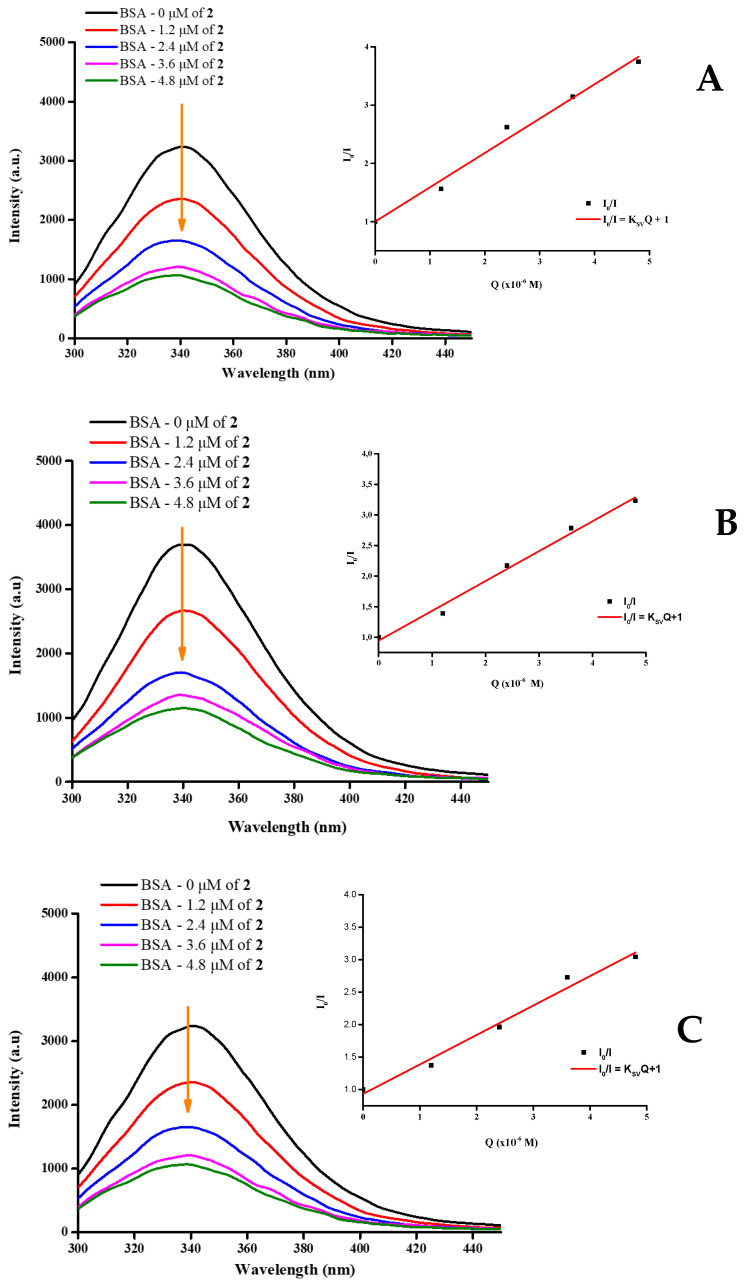
Fluorescence spectra of solutions containing BSA (1.5 μM, PBS) and molar ratios of **2** (DMSO). **Inset**: Stern–Volmer plot acquired from steady-state fluorescence at (**A**) 20 °C, (**B**) 30 °C, and (**C**) 37 °C.

Bovine serum albumin (BSA) exhibits an emission band at 340 nm, when in the excited state. Specifically, BSA contains two tryptophan residues: Trp 134, situated on the hydrophilic surface, and Trp 213, located in the hydrophobic region of domain IIA [50]. The incorporation of metal-containing complex compounds leads to a noticeable quenching effect, as illustrated in Figure 12 for **2**, and Figures S12 and S13 for **1** and **3**, respectively.

This quenching phenomenon, attributable to alterations in the environment around the protein tryptophan residues, substantiates the interaction between serum albumin and the title lanthanide compounds. The aforementioned interaction has already been confirmed through UV-visible spectroscopy.

To quantify the observed effects, the Stern–Volmer constants as well as the quenching rate constants can be ascertained via application of the Stern–Volmer equation (Equation (1)). To that end, the Stern–Volmer constant delineates the gradient within the linear regression analysis, while determination of the quenching rate constant is facilitated via Equation (2) (Figure 12, Figures S12 and S13). Furthermore, utilization of the Scatchard plots (Figures S14–S16) and the corresponding equation (Equation (3)) enables computation of both binding constants and quantitation of the number of binding sites. In Equation (3), n signifies the gradient of the linear regression and K_b_ denotes the intercept represented as an exponent of ten. The outcomes of the measurements for **1**–**3** across the three different temperatures are presented in Table 3 and Table 4.

**Figure 13:**
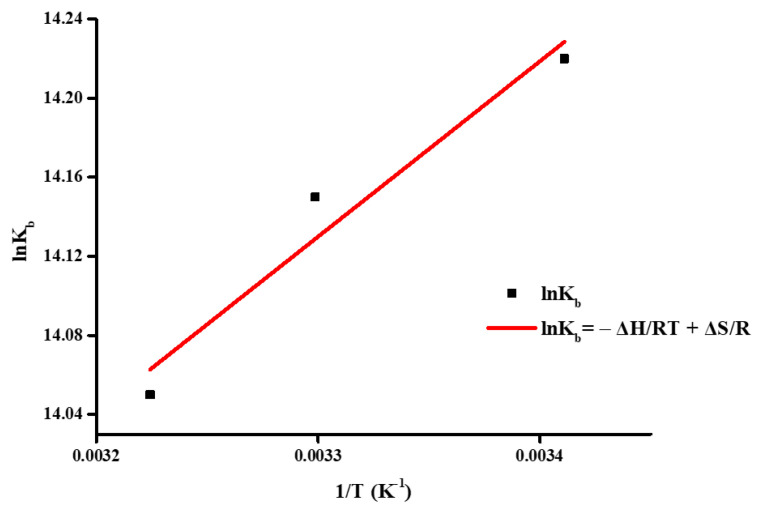
Van’t Hoff plot of **2** from measurements at 20 °C, 30 °C, and 37 °C.

With regard to the attributes of the binding forces, determination of enthalpy, entropy, and Gibbs energy values can be attained through examination of the Van’t Hoff plot, as depicted in Figure 13 and Figure S17 through Equations (4) and (5). Herein, the enthalpic variation manifests as the slope, while the intercept signifies the entropic alteration.

#### 2.10.3. Circular Dichroism

**Figure 14:**
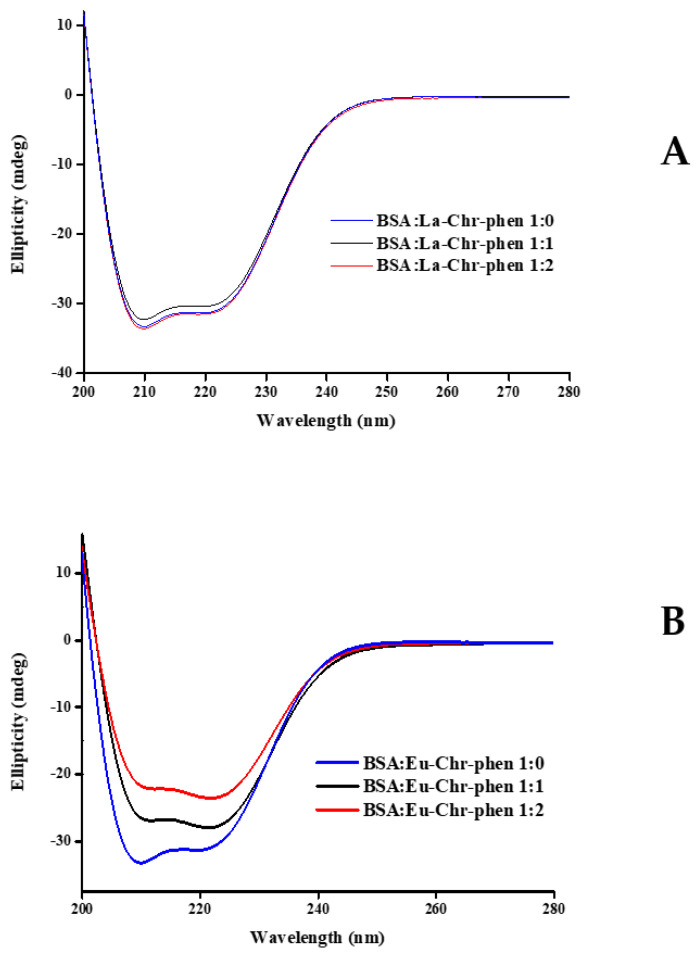
Circular dichroism spectra of solutions containing BSA (1 μM, PBS) and increasing molar ratios of (**A**) **1** (MeOH) and (**B**) **3** (MeOH).

Circular dichroism measurements were performed to examine the influence of the materials on the protein secondary structure. As depicted in Figure 14, two bands are observed at 208 and 222 nm.

Upon addition of the compounds to the BSA solution of constant concentration (1.0 μM), in the case of material **1**, the spectra exhibit no significant variation upon mixing with BSA (Figure 14A). In the case of material **3**, a substantial reduction in the intensity of the aforementioned bands was noted, thus indicating a decrease in the α-helical content (Figure 14B). The analytical determination of the α-helical content (%) in the protein, which was obtained through utilization of Equations (6) and (7), is presented in Table 5. Circular dichroism spectra of **2** were not obtained due to solubility restrictions in methanol.

#### 2.10.4. Molecular Docking

Ligand docking studies were conducted to investigate the interaction of compounds **1**, **2**, and **3** with BSA, using the energy-minimized crystal structure of BSA (PDB ID: 6QS9), highlighting specific residues involved in ligand–protein interactions within different subdomains of the protein. The computed binding/docking energies for the best poses of **1**, **2**, and **3** at the BSA binding sites are −11.2, −10.4, and −10.4 kcal/mol, respectively.

**Figure 15:**
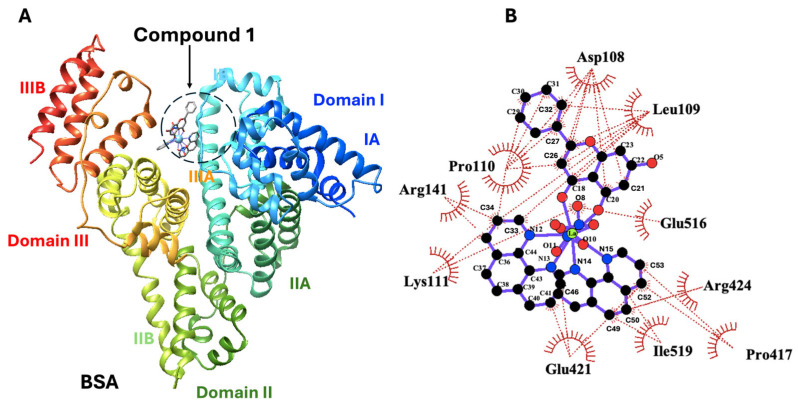
(**A**) Compound **1** was docked against the 3D structure of BSA. (**B**) 2D interaction diagrams illustrate the interactions between compound **1** and the BSA binding motif (hydrophobic contacts are indicated in red).

Compound **1** interacts with a region bridging subdomains IB and IIIA/IIIB of BSA, establishing hydrophobic contacts with specific residues: Asp108, Leu109, Glu516, Arg424, Pro417, Ile519, Glu421, Lys11, Arg141, and Pro110 (Figure 15). Notably, Asp108, Leu109, and Pro110 in subdomain IB interact with the benzyl and heterocyclic pyran rings of the Chr group of the ligand. Residues Glu421, Arg424, and Pro417 in subdomain IIIA interact with the central benzene and pyridine rings of phen, while Glu516 and Ile519 in subdomain IIIB make contacts with NO_3_ and phen, respectively. In addition, Lys11 in domain I and Arg141 in subdomain IIA interact with the pyridine ring of phen.

Compound **2** exhibits a primary binding affinity for subdomain IIA of BSA, with secondary interactions extending into subdomains IB and IIIA (Figure S18A). It forms hydrophobic contacts with residues Asp105, Ala197, Lys201, Glu461, Thr474, Gln200, Ser101, and Lys103 (Figure S18B). Notably, Asp105, Lys103, and Ser101 in subdomain IB interact with the benzyl ring of Chr and phen groups of the ligand. Residues Gln200, Ala197, and Lys201 in subdomain IIA are involved in contacts with the pyridine ring of phen and the benzene ring of Chr, respectively. Arg193 in subdomain IIA forms a hydrogen bond between NH1 and the phenolic group of the benzene ring of Chr (Figure S18B), while Glu461 and Thr474 in subdomain IIIA interact with the benzene and pyran rings of the Chr group of the ligand.

Compound **3** primarily binds to subdomain IIA of BSA, with significant interactions in subdomain IB and additional contacts in subdomain IIIA (Figure S19A). It establishes hydrophobic contacts with residues Lys201, Gln200, Glu461, Arg193, Val458, Lys103, Asp104, Lys642, Asp105, Ser101, and Gln200 (Figure S19B). Specifically, residues Ser101, Asp104, Asp105, and Lys103 in subdomain IB interact with the benzene rings of phen and Chr groups of the ligand. Residues Gln200, Lys201, and Arg193 in subdomain IIA interact with the benzyl and heterocyclic pyran ring of Chr. Tyr144 in subdomain IIA forms a hydrogen bond with the benzene ring of Chr through their phenolic groups (Figure S19B). In subdomain IIIA, Val458, and Glu461 interact with the benzene rings of both Chr groups, while Lys642 makes contacts with the benzene ring of Chr.

### 2.11. In Vitro Antimicrobial Activity

The antimicrobial efficacy was studied at different concentrations, expressed as mass of compounds (mg) in a total mass value of disc containing 30 mg (Compound + LB Agar). Compounds **1**–**3** were mixed at different proportions with LB Agar, and the concentrations studied are shown in Table S2. For the antimicrobial efficacy of the title compounds **1**–**3**, the minimum inhibitory concentration (MIC) was determined in each case on an agar plate using the disc diffusion method (Figures S20 and S21). In Table S2, the MIC values (mg) are shown for each compound in two different bacterial cultures. For compound **1**, an MIC value of 0.05 mg was shown for *E. coli* and 10 mg for *S. aureus*. The corresponding ZOI values were 20.0 ± 0.1 mm and 15.5 ± 0.1 mm, respectively. Compound **2**, exhibited a ZOI value of 28.5 ± 0.1 at a concentration of 1 mg in the case of *E. coli* and 16.5 ± 0.1 at 10 mg in the case of *S. aureus*. Compound **3** at a concentration of 10 mg exhibited ZOI values of 22.8 ± 0.1 mm and 20.5 ± 0.1 mm for *E. coli* and *S. aureus*, respectively. The inhibitory concentration of the compounds was compared to that of free Chr, phen, and metal salt. In the case of metal salts and free flavonoids, there was no ZOI observed in both bacteria, except for Chr. The latter material exhibited a ZOI value of 29.0 ± 0.1 mm at very high concentrations. The ZOI of phen is greater than 30.0 mm.

## 3. Discussion

### 3.1. Synthetic Challenges in Lanthanide-Flavonoid Chemistry

The concept of synthesizing hybrid metal–organic materials involving flavonoids and lanthanide ions emerges from the (a) antioxidant properties attributed to flavonoids and (b) the prospect of modulating lanthanide chemical reactivity alongside flavonoid antioxidant potency, thus giving rise to biologically competent and potentially enhanced bioactivity agents. Cognizant of the chemical reactivity of lanthanides and the chemically justified structural features of flavonoids, the present research pursued the synthesis of binary–ternary complex assemblies, bearing these two essential components (and third ancillary chelator ligands contributing to their stability) in varying molecular ratios and structural formulations.

In that respect, for the synthesis of all ternary complexes described in this work, lanthanide ions Ln(III) [La(III), Nd(III), and Eu(III)] reacted with a structurally simple flavonoid, Chr, through the C4 ketonic group of the pyrene ring and the C5 phenolic group of the A ring, in the presence of the ternary N,N-aromatic chelator phen. In addition, triethylamine was utilized, due to its nature as a Lewis base, to elicit deprotonation of the Chr phenolic moiety, thereby triggering binding reactivity of the flavonoid to the metal ions. In all cases, chemical reactivity exemplified under solvothermal reaction conditions led to the formation and isolation of ternary Ln-flavonoid-phen crystalline materials **1**–**3**. The physicochemical characterization of all three complex materials was pursued through elemental analyses, spectroscopic techniques (FT-IR, UV-visible, luminescence, ESI-MS, magnetic susceptibility, Mössbauer), accompanied ultimately by X-ray crystallography. The three-dimensional crystal structure of the materials validated their analytical and spectroscopic studies, thus providing grounds for further evaluation of their biological reactivity (vide infra). To that end, the composition of the coordination sphere (composition, coordination number) of the lanthanide ions, combined with the mode of ligand binding (flavonoid, phen, nitrate anion), provided a clear picture of the assembly process associated with the structural integrity of the compounds themselves. Outstanding in the characterization of the title compounds were the magnetic susceptibility studies that provided the fingerprint behavior in each case, commensurate with the identity (electronic and structural) of the lanthanide ions participating in the arisen complex assemblies. Furthermore, the ^151^Eu Mössbauer spectroscopic investigation of compound **3** provided a rare glimpse of the specific nucleus, thus attesting to the identity of the coordination sphere of one of the very few examples of such materials and their spectroscopic signature provided by experimental γ-absorption studies.

Furthermore, experimental observations pertaining to the nature of the complex species **1**–**3** were ascertained through theoretical calculations, involving (a) bond valence sum (BVS) calculations pinpointing the oxidation state of the lanthanide ion, and (b) Hirshfeld analysis, seeking answers to questions involving intra-/intermolecular interactions in the solid state. To that end, Hirshfeld surface analysis and fingerprint plots were employed for the determination of the complex structures in **1**–**3** by interpreting intermolecular interactions throughout all three species architectures. In such a context, normalized contact distances, curvedness, and shape index surfaces contribute to the understanding of different intermolecular interactions within each crystal structure of the materials produced. The overall amassed information helps formulate in a rational manner the properties that could contribute to understanding potential interactions with bioavailable molecular targets in biological fluids.

Based on the assertions on the spectroscopic signature in the solid state, solution works (UV-visible and ESI-MS) were key to defining the overall physicochemical profile of the three compounds. That turned out to be important in further supporting the investigation of the purported interactions of the species in solution when biomolecular targets such as bovine serum albumin (BSA) are involved (vide infra). This work prompted further investigation toward biological experiments that delve into the biochemical and biological activity of the title compounds, thereby exemplifying the roles linked to the actual function of the synthesized materials.

### 3.2. Structural Variations in Lanthanide-Flavonoid Assemblies

The chemical reactivity of the investigated ternary systems leads to structures of the derived lanthanide–Chr–phen complex assemblies, which draw attention with respect to the composition of the coordination sphere and the disposition of the participating partners. i.e., the lanthanide ion, Chr, and the nitrate ion; the latter moiety is poised to occupy coordination sites in the metal assembly or abstain from coordination yet plays the role of a counter ion.

To that end, what is observed in all cases is that in the process of metal binding to Chr, stabilization of the emerging complex assemblies is achieved through the formation of a six-membered chelate ring involving the metal ion and the flavone, and a five-membered ring formulated by the metal ion and the sp^2^ nitrogen terminals of the phen ligand. The adopted ligand-binding modes are similar to the ones observed in reported lanthanide compounds bearing β-diketonate moieties [51,52,53]. As a result, the coordination number for complex assembly **1** is 10, whereas the coordination number for **2** and **3** is 8, both similar to the aforementioned compounds reported in the literature [51,52,53]. To that end, the metal ion–Chr ratio in **1** is 1:2, in contrast to **2** and **3**, where the same ratio is 1:1. Examination of the structures of the three compounds shows that the coordination sphere compositions of the metal ions in **1** (La) and **2** (Nd) are distinctly different from that in **3** (Eu). Compared to the above mentioned compounds [51] in the literature, compound **2** shows a similar coordination with four bound ligands (two molecules of Chr and two phen aromatic chelators). More specifically, the complex assembly in **1**, with La being the first member of the lanthanide series, shows that the coordination sphere consists of one flavonoid ligand, two phen molecules, and two bound nitrato ligands. Moving on to the right, the immediately abutting lanthanide complex assembly in **2** projects a different environment in which there exist two flavonoid and two phen ligands. It seems as if the two nitrato ligands in **1** have been displaced by one flavonoid ligand, the bulk structural constraints of which satisfy the coordination environment of the assembly in **2**, with the nitrate ion staying outside the coordination sphere and acting as a counterion. In the case of **3**, the distribution pattern of the ligands in the coordination sphere around the Eu(III) center reveals the presence of one phen and one nitrato ligand, with two flavonoids bound to the metal ion center

Comparison to previously characterized lanthanide complexes with Chr [29] reveals the following: (a) the presence of both one phen and one nitrato ligands appears to be a pattern applying to the coordination sphere of Sm(III), Eu(III), Dy(III), and Er(III) species; (b) the presence of two flavonoids, one phen ligand, and one nitrato ligand appears to be a pattern applying to the coordination sphere of Eu(III), Dy(III), and Er(III) species; and (c) the exception to the rule is the Sm(III) complex assembly, with one flavonoid, concurrently with the common pattern of one phen and one nitrato ligand described above. By analogy to the herein described 4f Ln(III) complexes bearing Chr, a meaningful comparison with the corresponding ternary complex species of other 3d metal ions, such as Cr(III) [54], reveals (a) higher coordination numbers for the lanthanide species, (b) similar bidentate chelating modes of the flavonoid and ancillary ligands bound to the metal ions in both Ln(III) and Cr(III) species, and (c) water ligands introduced into the coordination sphere of Cr(III) that do not appear in the coordination sphere of Ln(III) species. The latter case is still being explored to identify reasons for which that happens, in view of the fact that the bound nitrato ligands appear in both types of metal coordination sphere.

The aforementioned trends emerging from the examination of the coordination sphere of the ternary lanthanide assemblies suggest the following: (a) the structural speciation of the ternary systems involving the flavonoid Chr, phen, and nitrate ions is quite diverse, with species emerging through careful selection of the reaction conditions and molecular stoichiometry of the starting reagents; and (b) the structural attributes of the complex assemblies derived so far provide a more diversely distributed ligand environment in the early part of the lanthanide series, slowly changing into numerical ligand combinatorial patterns that might be related to physical parameters of the elements in the lanthanide series (e.g., size of trivalent ions, chemistry, etc.) [29]. To that end, currently ongoing efforts in the laboratory are devoted to exploring and delineating the aforementioned contentions.

### 3.3. Luminescence and Magnetic Properties

Apart from their aforementioned properties, all materials were scrutinized with respect to their optical properties, through luminescence, and their magnetic susceptibility behavior. Luminescence studies indicated the occurrence of quenching in all cases of materials, for both phen and Chr, when bound to the metal ion, in comparison to the free ligand(s), similar to previous findings involving ternary Ln(III) [29] and Cr(III) compounds containing bound flavonoids [54]. It is worth pointing out at this juncture that there were no f–f transitions observed in the spectra of **1**–**3**, in line with previous findings [29] (except for Sm(III)) and molecules [51,52,53] exhibiting similar structural assemblies. In that respect, the newest family of species possesses luminescent properties that (a) exemplify the electronic structure and hence the optical activity observed in previous cases of analogous complex assemblies within the lanthanide series, and (b) set the basis for further correlation with other physical properties, such as those linked to the magnetism of the species.

In order to investigate the magnetic behavior of materials **1**–**3**, their magnetic susceptibility was examined as a function of temperature, while magnetization as a function of temperature concurrently extended the picture of the three materials in a conclusive manner. To that end, the diamagnetism for La(III) and the paramagnetic behavior of Nd(III) and Eu(III) signify the observed behavior, with the detailed picture intimately associated with the partial occupation of the intervening f orbitals with electrons, collectively configuring the magnetic make-up of the solid state assemblies. Undoubtedly, such a collection of magnetic profiles could be linked to the ascertained fact that the luminescent properties of **1**–**3** call for quenching, thereby suggesting correlations in the magneto-optical realm, serving as potential sensors in abiotic and/or biological applications.

### 3.4. Biomolecular Interactions with BSA

In the context of investigating the potential biological activity of the title compounds in aqueous media, the interactions of the complexes with a known binder and carrier of molecules, i.e., BSA, were examined. Initially, UV-visible spectroscopic studies perusing the interactions of **1**–**3** with BSA led to the observation that conformational changes occur in BSA following the interactions with the title complexes. The changes incurred were evident in the spectral absorption features, in which absorbance variability was observed in line with the perturbation of the environment of aromatic residues. The ascertained spectral changes in the UV-visible spectra of the binary BSA–complex systems are consistent with changes in the α-helical content of the protein. Further examination of the involved interactions through luminescence led to the realization that upon increasing concentrations of the title complexes interacting with BSA, the quenching effect progressively increases. Quantification of the observed effect was pursued through determination of the Stern–Volmer equations. To that end, the emerging rate constants for quenching exceed the value of 10^10^ M^−1^ s^−1^, as shown in Table 3, thereby signifying the presence of the lowest limit for a static quenching mechanism. This, in turn, suggests that the compounds under examination form an adduct in the ground state via a static quenching mechanism [55]. The number of binding sites n was subsequently determined through Scatchard plots. The plots show that n is close to 1, which indicates the stoichiometric composition of 1:1 for the complex between BSA and the title compound(s). The high binding constant values of K_b_ indicate that the compounds can effectively bind to the protein and be transferred to the potential biological target. To that end, these values are significantly lower than 10^15^ M^−1^, which is the strongest known non-covalent interaction. As a result, it appears that the title compounds can reversibly bind to the bovine serum albumin and be released upon arrival to the target. Moreover, data pertaining to thermodynamics have been utilized to ascertain the characteristics of the forces engaged in the investigated interactions (Table 4). Binding experiments were conducted at three different temperatures to determine the values of enthalpy and entropy changes, along with the calculation of the Gibbs free energy. The observed positive changes in both enthalpy and entropy in the case of the complex compounds of La(III) (**1**) and Eu(III) (**3**) imply that hydrophobic bonds primarily drive these interactions. On the other hand, the Nd(III) complex **2** showcases negative enthalpy changes coupled with positive entropy changes, thus suggesting predominance of electrostatic interactions. Moreover, the presence of negative Gibbs energy values, at all temperatures studied, substantiates the spontaneous nature of the interaction between protein and the title compounds [56]. The UV-visible results are further substantiated by circular dichroism measurements. Comparative analysis of these two characterization techniques revealed that a greater reduction in the intensity of the circular dichroism spectra corresponds to a more significant decrease in the absorbance of the UV-visible spectra, both translating into a major differentiation of the protein’s secondary structure.

### 3.5. Docking Studies Against BSA

The ligand docking studies reveal the distinct binding patterns and interactions of compounds **1**, **2**, and **3** with BSA, each showing unique preferences for specific subdomains of the protein. Compound **1** demonstrates the strongest binding affinity, with a computed binding energy of −11.2 kcal/mol, primarily interacting with subdomains IB, IIIA, and IIIB of BSA. It forms hydrophobic contacts with residues, such as Asp108, Leu109, Glu516, and Arg424 through its benzene and heterocyclic pyran rings of Chr and phen groups. Notably, interactions with residues in subdomain IB (Asp108, Leu109, Pro110) highlight a bridging interaction between subdomains IB and IIIA/IIIB. Compound **2** exhibits a slightly lower binding energy of −10.4 kcal/mol, with its primary interaction centered on subdomain IIA of BSA. It extends its contacts into subdomains IB and IIIA, forming hydrophobic interactions with residues such as Asp105, Ala197, Lys201, and Glu461. Noteworthy interactions include a hydrogen bond between Arg193 and the phenolic group of Chr’s benzene ring in subdomain IIA, thus highlighting specific interactions with both Chr and phen rings of the ligand across different subdomains. Similarly, compound **3** also demonstrates a binding energy of −10.4 kcal/mol, primarily binding to subdomain IIA, but with significant interactions in subdomains IB and IIIA as well. It engages residues like Lys201, Gln200, and Glu461 through hydrophobic contacts with the Chr’s benzene and pyran rings. Notably, a hydrogen bond is observed between Tyr144 and the phenolic group of Chr’s benzene ring in subdomain IIA, thus indicating a specific interaction mode unique to compound **3**.

Overall, compounds **1**, **2**, and **3** show comparable binding energies. The distinct residues engaged in interactions vary among the compounds, emphasizing their individual binding modes and preferences within the BSA structure. These observations underscore the structural elements and potential that enable these compounds to selectively interact with diverse regions of BSA, suggesting potential implications for their bioactivity profile.

As demonstrated by the docking results, Chr molecules play a pivotal role in binding to BSA, with the majority of the compound’s interactions with amino acid residues originating from the Chr pyran ring. These theoretical findings are largely consistent with previously mentioned experimental data (vide supra). Metal ions coordinated with two Chr molecules exhibited a stronger binding constant and induced greater alterations in the α-helical content compared to the material containing a single flavonoid molecule. However, experimental data diverge from the docking results concerning the binding affinity of the synthesized materials. Furthermore, the docking results align with the findings from luminescence measurements. Specifically, materials **2** and **3**, which are shown in docking simulations to be closer to Trp134 and Trp213, respectively, exhibited a more pronounced quenching phenomenon than **1**.

### 3.6. In Vitro Antibacterial Profile

The antibacterial efficacy in the present study was investigated through the disc diffusion method in Gram(−) (*E. coli*) and Gram(+) (*S. aureus*) bacterial strains. The efficacy of inhibition was measured by ZOI values and reflected into the MIC values for every compound through a concentration-dependent study. All studied compounds **1**–**3** have been compared, in terms of their antibacterial efficacy, with the free flavonoid, the aromatic chelator, and metal salts (all expressed as the same amount on a molar scale, based on their molecular structure). Comparison of the results from both types of bacteria reveals that the MIC value for **1**, in the case of *S. aureus*, is 200 times higher than that of *E. coli*, thus suggesting that the La(III)-based hybrid material exhibits higher antibacterial activity in *E. coli* than in *S. aureus*. In the case of the Nd-Chr-phen hybrid material, the MIC value is 10 times higher in *S. aureus* compared to that in *E. coli*. Compound **3** (Eu-Chr-phen) exhibits the same MIC value in both bacteria. Following the comparison of the antibacterial activity of the studied compounds with that of free ligands (Chr, phen) and metal salts, it appears that Chr and appropriate metal salts have no or lower antibacterial properties than those of the hybrid compounds, except for the phen chelator, which shows very high activity. All compounds have shown enhanced antibacterial activity due to their structure-specific architecture and the formulation of their composition. In the case of *E. coli*, compounds **1** and **2**, due to the fact that they bear two phen aromatic chelators in their structure, show antibacterial activity with lower MIC values. Differentiation of that behavior occurs in compound **1**, which exhibits the lowest value of MIC value, thus verifying the notion that the combination of the specific metal ion (La(III)) with Chr and phen in the specific coordination complex assembly facilitates traversal of the bacterial membrane based on the complex assembly’s specific properties. Moreover, compound **3**, with one phen and one Chr moiety in its coordination sphere, exhibits the highest MIC value. To that end, the overall antibacterial profile of the compounds is structure-specific, and selectivity against Gram(−) bacteria is related to the overall structure architecture of the hybrid materials.

## 4. Experimental

### 4.1. Materials and Methods

The selection of lanthanide metal salts includes Neodymium(III) nitrate hexahydrate (Nd(NO_3_)_3_·6H_2_O, 99.9%), purchased from Alfa Aesar, (Alfa Aesar, Ward Hill, MA, USA), with Lanthanum(III) nitrate (La(NO_3_)_3_) and Europium(III) oxide (Eu_2_O_3_) both provided by Fluka (Honeywell International Inc., USA). Chrysin C_15_H_10_O_4_, >98.0%, (Chr) was purchased from TCI (Tokyo Chemical Industry Co., Ltd., Tokyo, Japan) and 1,10 phenanthroline (C_12_H_8_N_2_, 99.0%, phen) as well as Dimethyl sulfoxide ((CH_3_)_2_SO, DMSO) were acquired from Sigma Aldrich (Sigma-Aldrich chemical company, Steinheim, Germany). Furthermore, triethylamine (C_6_H_15_N) was provided by Carlo Erba (Carlo Erba Reagents GmbH, Emmendingen, Germany). Moreover, bovine serum albumin was purchased from Merck. Finally, LC-MS grade methanol (99.9%) and (CH_4_O) nitric acid 65% were provided by Chem-Lab (Chem-lab NV, Zedelgem, Belgium) and VWR Chemicals (VWR International Ltd., Radnor, PA, USA), respectively.

**Note:** Europium(III) nitrate hydrate was prepared from Europium(III) oxide according to the literature, introducing slight modifications [57]. The synthesis and FT-IR spectra [58] are shown in Figure S1.

### 4.2. Physical Measurements

Infrared spectra were recorded using a Nicolet FT-IR 200 spectrometer (Thermo Fisher Scientific Inc., Waltham, MA, USA) at room temperature conditions using KBr pellets.

A Hitachi U-1900 spectrophotometer (Hitachi Ltd., Tokyo, Japan) was used to carry out the UV-visible measurements of all the solutions prepared within the range of 190–1100 nm. The SYSTAT Inc. peakfit (version 4.11) program (Chicago, IL, USA) was employed for the spectral fitting of the lanthanide complex solutions. The fitting process was based on the Savitzky–Golay algorithms, using (a) number of iterations 8000–10,000, (b) number of significant digits 6, and (c) full curvature matrix, until R^2^ indicated a value of 0.9999 ± 0.0001.

A ThermoFinnigan Flash EA 1112 CHNS elemental analyzer (Thermo Fisher Scientific Inc., USA) was used for the quantitative determination of carbon, hydrogen, and nitrogen. The analyzer operation relies on the dynamic flash combustion of the investigated sample (at 1800 °C), followed by reduction, trapping, complete GC separation, and detection of the products. The instrument is fully automated and monitored via the Eager 300 dedicated software (version 1).

#### 4.2.1. ESI-MS

Electrospray ionization mass spectrometry (ESI-MS) infusion experiments were carried out on a Thermo Fisher Scientific model LTQ Orbitrap Discovery MS (Bremen, Germany) in methanol. Solutions of compounds **1** and **3** with molecular formulae M_1_ = [La(C_15_H_9_O_4_)(C_12_H_8_N_2_)_2_(NO_3_)_2_]·1.5CH_3_OH and M_3_ = [Eu(C_15_H_9_O_4_)_2_(C_12_H_8_N_2_)(NO_3_)] were introduced into the ESI source of the MS at a flow rate of 5 μL/min using an integrated syringe pump. The infusion experiments were conducted using a standard ESI source operating in a positive ionization mode. The operating conditions of the source were as follows: 3.7 kV spray voltage and 320 °C heated capillary temperature.

#### 4.2.2. Photoluminescence

A Hitachi F-7000 fluorescence spectrophotometer from Hitachi High-Technologies Corporation (Hitachi Ltd., Japan) was used for the investigation of the luminescence activity of compounds **1**–**3**. Emission (em) and excitation (ex) spectra were recorded with split widths of 5.0 nm and a scan speed 1200 nm·min^−1^. Measurements were pursued at room temperature in the solid state. FL Solutions 2.1 software (version 2.1) was employed, supporting the entire system on Windows XP.

#### 4.2.3. Magnetic Susceptibility Studies

The powdered polycrystalline samples of the title compounds were placed in polypropylene capsules and their magnetic properties investigated using the vibrating sample magnetometer (VSM) option of a physical property measurement system (PPMS) by Quantum Design (Pfungstadt, Germany). The capsules were inserted into a brass sample holder, which was attached to the sample holder rod of the VSM. The samples were then investigated in a temperature range from 3 to 300 K using measurements of M(T,H), with applied fields of up to 80 kOe. Data for the neodymium and europium samples were corrected for the sample holder and the intrinsic diamagnetism, based on the increment list by Bain and Berry [59]. Fitting and plotting of the data were performed using OriginPro 2016G (version 9.3.2.303) [60]. Graphical editing took place through the program CorelDRAW2017 (version 19.0.0.328) [61].

#### 4.2.4. ^151^Eu Mössbauer Spectroscopy

A ^151^Sm:EuF_3_ source was used for the Mössbauer spectroscopy experiment on [Eu(C_15_H_9_O_4_)_2_(C_12_H_8_N_2_)(NO_3_)]. The measurement was conducted at 78 K (standard liquid nitrogen bath cryostat) in usual transmission geometry, while the source was kept at room temperature. About 30 mg of the sample was placed in a thin-walled PMMA container with a diameter of 15 mm. Fitting of the data was performed using the WinNormos for Igor7 program package (version 7.010) [62].

### 4.3. Theoretical Calculations

Bond valence sum (BVS) calculations were recorded using the Visualization for Electronic and Structural Analysis (VESTA) program (Version 3.4.8) [63]. All calculations were obtained through the empirical formula S = exp[(R_o_ − r)/b], where b = 0.37 Å in all cases, r is the observed bond length, and R_o_ (Å) is a tabulated constant. In the case of La(III), Nd(III), and Eu(III) metal centers, specific R_o_ values were selected from the literature as follows: La(III)-O 2.148, La(III)-N 2.261, Nd(III)-O 2.086, Nd(III)-N 2.201, Eu(III)-O 2.038, Eu(III)-N 2.161 [64,65].

### 4.4. Hirshfeld Surface Analysis

The Crystal Explorer (version 21.5) [66] and Inskape 1.0.1 programs were employed for the generation of the Hirshfeld surface analysis [67,68,69] and two-dimensional (2D) fingerprint plots [70,71,72]. During mapping, bond lengths to hydrogen atoms were set to standard values [73].

In all three compounds studied, the electron density boundary surfaces between molecules in a crystal structure were drawn out, thereby allowing for the presentation and interpretation of various intermolecular interactions. During the conducted analysis, an emerging isosurface is obtained, while each point of the isosurface can be defined by two distances: (a) d_e_, the distance from the point to the nearest atom outside the surface, and (b) d_i_, the distance to the nearest atom inside the surface.

### 4.5. Synthesis

**Synthesis of La-Chr-phen [La(C_15_H_9_O_4_)(C_12_H_8_N_2_)_2_(NO_3_)_2_]·1.5CH_3_OH (1)**. In a round-bottom flask, a mixture of 14 mL methanol/water (1/1 *v*/*v*) was added to dissolve solid La(NO_3_)_3_ xH_2_O (0.055 g, 0.17 mmol) under continuous stirring. Subsequently, the addition of Chr (0.045 g, 0.18 mmol) changed the color of the solution from colorless to cloudy yellow. No further color change was observed upon addition of phen (0.029 g, 0.16 mmol). Subsequently, triethylamine (0.023 mL, 0.17 mmol) was added dropwise to the reaction mixture and the color turned clear yellow. The final reaction mixture was transferred to a Teflon-lined stainless-steel reactor (23 mL) and heated to 100 °C for 2 h. Plate yellow crystals were isolated by filtration. Yield 0.097 g (59%). Anal. Calcd. for **1**, (C_40.5_H_31_LaN_6_O_11.5_ M_r_ = 924.63): C, 52.56; H, 3.35; N, 9.08. Found: C, 52.51; H, 3.32; N, 9.05.

**Synthesis of Nd-Chr-phen [Nd(C_15_H_9_O_4_)_2_(C_12_H_8_N_2_)_2_](NO_3_)·0.5CH_3_OH (2)**. In a round-bottom flask containing 7 mL of water and 7 mL of methanol, Nd(NO_3_)_3_·6H_2_O (0.11 g, 0.25 mmol) was added under continuous stirring until the solution became clear and transparent. To that, 0.064 g (0.25 mmol) of Chr was added and the solution turned cloudy yellow. Subsequently, addition of phen (0.045 g, 0.25 mmol) and triethylamine (0.035 mL, 0.25 mmol) resulted in no color change. The final reaction mixture was transferred to a Teflon-lined stainless-steel reactor (23 mL) and subjected to a solvothermal process at 140 °C for 48 h. Following gradual cooling of the reaction mixture to room temperature, pale green bar crystals were isolated by filtration. Yield: 0.10 g (~38%) Anal. Calcd. for **2** (C_54.5_H_36_NdN_5_O_11.5_ M_r_ = 1089.15): C, 60.05; H, 3.31; N, 6.43. Found: C, 60.01; H, 3.29; N, 6.41.

**Synthesis of Eu-Chr-phen [Eu(C_15_H_9_O_4_)_2_(C_12_H_8_N_2_)(NO_3_)] (3)**. A quantity of 0.11 g (0.25 mmol) of Eu(NO_3_)_3_∙xH_2_O was dissolved in a 14 mL solution of methanol and water (1:1 *v*/*v*) in a round-bottom flask under continuous stirring. Addition of Chr (0.13 g, 0.50 mmol) caused a color change from clear to cloudy yellow. Further addition of phen 0.045 g (0.25 mmol) and triethylamine 0.035 mL (0.25 mmol) did not significantly change the color of the solution. Finally, the reaction mixture was heated at 140 °C, for 48 h, in a Teflon-lined stainless-steel reactor (23 mL). At the end, orange crystals were recovered using filtration. Yield: 0.12 g (54%) Anal. Calcd. for **3**, (C_42_H_26_EuN_3_O_11_ M_r_ = 900.64): C, 55.96; H, 2.89; N, 4.66. Found: C, 55.93; H, 2.88; N, 4.62.

### 4.6. X-Ray Structural Determination

Single crystals of all three compounds, suitable for crystallographic investigation, were mounted at room temperature (**3**) or at 130 K (**1**, **2**) on a Bruker Kappa APEX2 (Bruker, Karlsruhe, Germany) diffractometer equipped with a Triumph monochromator and a Kryoflex II cooler system using Mo Kα (λ = 0.71073 Å, source operating at 50 kV and 30 mA) radiation. Unit cell dimensions were determined and refined by using the angular settings of at least 163 high-intensity reflections (>10σ(I)) in the range 10° < 2θ < 20°.

Intensity data were recorded using φ and ω–scans. All single crystals exhibited no decay during data collection. The frames collected were integrated with the Bruker SAINT Software package (version 2) [74] using a narrow-frame algorithm. Data were corrected for absorption using the numerical method (SADABS), which is based on crystal dimensions [75]. The structures were solved using SUPERFLIP (31 August 2007) [76] incorporated in Crystals. Data refinement (full–matrix least–squares methods on F^2^) and all subsequent calculations were carried out using the Crystals version 14.61 build 6236 program package [77]. All non–hydrogen non-disordered atoms were refined anisotropically. For the disordered atoms, their occupation factors were first refined under fixed isotropic parameters. Their anisotropic/isotropic displacement factors were finally refined under previously observed and normalized fixed occupancy factors.

Hydrogen atoms bonded to non-disordered atoms were located from difference Fourier maps and refined using soft constraints at idealized positions riding on the parent atoms with isotropic displacement parameters U_iso_(H) = 1.2U_eq_(C) and 1.5U_eq_ (–OH hydrogens) at distances C––H 0.95 Å and O–H 0.82 Å. All OH hydrogen atoms were allowed to rotate. For the disordered hydrogen atoms with disordered parent atoms, their positions were selected to fulfil the previous demands as well as the hydrogen bonding demands, when necessary.

Crystallographic data for the complexes are presented in Table 1. Details on the conducted crystallographic studies as well as atomic displacement parameters are provided as Supporting Information in the form of cif files.

### 4.7. Interactions with Bovine Serum Albumin

The stability and interaction of complexes **1**–**3** with BSA were studied through UV-visible spectroscopy, tryptophan (Trp) luminescence quenching and circular dichroism. Pure dimethyl sulfoxide (DMSO) was utilized as a solvent for the dissolution of the synthesized materials during the UV-visible and luminescence measurements. In the case of Circular Dichroism measurements, the solvent used was methanol. In the case of the UV-visible measurements, a BSA (3 μΜ) solution was used in a phosphate buffer saline (PBS: 25.9 mM Na_2_HPO_4_, 1.78 mM KH_2_PO_4_, 2.74 mM KCl, 137 mM NaCl) solution. Measurements were conducted in the range from 200 nm to 400 nm and different absorption spectra were recorded as a function of increasing concentration of the investigated metal complex(es). In a similar fashion, luminescence quenching experiments were carried out using BSA (1.5 μΜ) solutions mixed with the title metal complexes in a gradually increasing molar ratio and studied in a temperature-dependent manner (25 °C, 30 °C, and 37 °C). Emission spectra were carried out throughout the range of 300–450 nm at an excitation wavelength of 280 nm. Results from the luminescence measurements were utilized in order to calculate the Stern–Volmer constant K_SV_, the quenching rate constant k_q_, and the binding constant K_b_. The number of binding sites n of the BSA–metal complex(es) was derived through the Stern–Volmer and Scatchard equations. Specifically, the K_SV_ and k_q_ constants were calculated using the Stern–Volmer equation [78]:

(1) $$\frac{I_{0}}{I}=1+K_{SV}\left[Q\right]=1+\tau_{0}k_{q}\left[Q\right]\;\;\;\mathrm{(Stern–Volmer)}$$

(2) $$k_{q}=\frac{K_{SV}}{\tau_{0}}$$

where I_0_ is the fluorescence intensity in the absence of the quencher, I is the fluorescence intensity in the presence of the quencher, [Q] is the concentration of the quencher, k_q_ is the quenching rate constant, and τ_0_ is the average lifetime of the fluorophore in the excited state (τ_0_ is usually 10^−8^ s for biomacromolecules [79]). On the other hand, K_b_ and n are calculated through linearization of the Hill equation, i.e., the Scatchard equation:

(3) $$\log\frac{\left(I_{0}-I\right)}{I}=\log K_{b}+n\log\left[Q\right]\;\;\;\mathrm{(Scatchard)}$$

where I_0_, I, and [Q] have the same meaning as in the previous equation, K_b_ is the binding constant, and n is the number of binding sites [79]. In addition, the thermodynamic parameters deciphering the nature of binding forces between the complexes and the protein can be determined through the Van’t Hoff equation:

(4) $$lnK_{b}=-\frac{\Delta H}{RT}+\frac{\Delta S}{R}\;\;\;\mathrm{(Van’t Hoff)}$$

The ΔH and ΔS values can be retrieved from linear regression of the lnK_b_ vs. 1/T plot. ΔG can be calculated through the following equation [79]:

(5) ΔG=ΔH−TΔS

In the case of circular dichroism measurements, a BSA solution at a concentration of 1 μM was employed. Spectral data were acquired over the range of 200 nm to 480 nm upon increasing concentration of the metal complex. The α-helical content of the protein was quantified using Equations (1) and (2).

(6) $$\left[\theta \right]=\frac{\theta_{\lambda }}{C_{p}\times n\times l\times 10}$$

(7) $$\alpha -helix\;\%=\frac{-\left[\theta \right]-4000}{33,000-4000}\times 100$$

where θ_λ_ is the measured ellipticity, C_p_ is the protein concentration, n is the number of amino acids, l is the path length, and [θ] is the mean molar ellipticity.

### 4.8. Docking Studies

The complex structures were constructed in three-dimensional coordinates, and their most stable conformation was determined through geometrical optimization, using the Spartan ’14 Molecular Modeling program (version 1.1.4) (Wavefunction Inc., Irvine, CA, USA). The initial structure of each molecule underwent energy minimization and conformational search, employing the Monte Carlo method with the MMFF94 molecular mechanics model within the Spartan ’14 suite. Further refinement to achieve the lowest energy conformer, in each case examined, was carried out via quantum-chemical calculations utilizing the ab initio Hartree–Fock method with a 6-31G* basis set. Molecular docking studies were conducted on the crystal structure of BSA (PDB ID: 6QS9). Docking simulations were performed using the AutoDock Vina open-source software (version 1.2.5) [80], through which compound–protein complexes were evaluated based on their energy scores and binding conformations. The best docked poses, characterized by lower binding energies and stronger interaction patterns, were selected from the docking results. UCSF Chimera (version 1.18.0) [81] was utilized for visualizing the molecular structures and analyzing the outcomes of the docking simulations, thus facilitating the construction of molecular models and visualization of molecular interactions.

### 4.9. In Vitro Antibacterial Properties

The in vitro antibacterial activity of **1**–**3** was investigated in Gram-positive (Gram (+)) (*Staphylococcus aureus*; *S. aureus*) and Gram-negative (Gram (−)) (*Escherichia coli*; *E. coli*) bacterial cultures in order to determine the standard zone of inhibition (ZOI). The disc diffusion method of lanthanide compounds was employed and pursued on autoclavable 25 mL Luria–Bertani agar (LB agar) (Applichem PanReac, Darmstadt, Germany) petri dishes of 90 mm [82]. The Luria–Bertani broth (LB broth) (Sigma Aldrich, Munich, Germany) was also used for the production of the inoculum, the negative control, and the positive control (penicillin-streptomycin (Biowest, Nuaillé, France)). All experiments were run in triplicates under aseptic conditions.

Specifically, prior to inoculation of bacteria in petri dishes, 3–5 freshly grown bacterial colonies were inoculated into a 3 mL LB broth, using a 25 mL Erlenmeyer flask in an Edmund Bühler TH15 shaking incubator (Edmund Bühler Gmb, Bodelshausen, Germany) at 37 °C for 1–2 h until the O.D. at 600 nm reached a value of 0.5 (5 × 10^5^ CFU·mL^−1^). Optical density (O.D.) measurements were carried out on a Hitachi UV-visible U-2800 spectrophotometer (Hitachi, Tokyo, Japan) to monitor the growth of bacteria in liquid cultures. Discs with a diameter of 60 mm containing LB agar and the tested materials totaling 30 mg in different concentrations were performed on petri dishes containing bacterial cultures and were further incubated for 12–15 h at 37 °C [83].

### 4.10. Statistical Analysis

All derived experimental data are presented as average ± SEM values of multiple sets of independent measurements. Mean cell survival rates and SEMs were calculated for each individual group. Absolute survival rates were calculated for each control group and one-way analysis of variance (ANOVA) was run for all pair comparisons, followed by post hoc analyses (Dunnett) using GraphPad Prism v.6 (GraphPad Software Inc., Boston, MA, USA). Significance levels were assessed as follows: * *p* < 0.05 (significant), ** *p* < 0.01 (highly significant), *** *p* < 0.001 (extremely significant), and **** *p* ≤ 0.0001 (extremely significant), or non-significant (*p* > 0.05).

## 5. Conclusions

Driven by the need to develop well-defined binary and ternary complex materials of lanthanides with flavonoids and ancillary aromatic chelators, the synthesis of three ternary metal–organic complexes, containing Chr, lanthanide ions Ln(III) [La, Nd, Eu], and an N,N’-aromatic chelator phen ligand, was pursued and achieved in methanol–water solvent media and in a concentration-dependent manner. In all cases, optimally determined solvothermal conditions led to the isolation of crystalline compounds. The physicochemical properties of the materials, both in the solid state and in solution, were characterized through analytical, spectroscopic, and X-ray crystallographic techniques, which were crucial in unraveling their identity. Additionally, theoretical calculations using BVS and Hirshfeld analysis enabled in-depth understanding of their properties. The luminescence activity of all three complex materials **1**–**3** showed quenching, thereby providing the basis for correlations with magnetic susceptibility work and use in future magneto-optical materials. Unique in that respect was the recording of the ^151^Eu Mössbauer spectrum of **3**, one of the rare occasions recorded in the literature. The collective physicochemical profile of **1**–**3** served as a preamble to the investigation of biological interactions with protein targets, such as BSA, and antimicrobial testing on Gram(−/+) bacteria. The experiments suggest that the intermolecular interactions involved are well-defined and thermodynamically driven, quantitatively exemplifying the observations made. Molecular docking calculations support the biological experiments and provide a broader picture of the title compounds, which lends credence to the notion that they can be used in further designing and synthesizing functional hybrid lanthanide–flavonoid materials of a defined structure-specific bioactivity as potential promoters of antimicrobial efficacy.

## Figures and Tables

**Table 1:** Crystal data and experimental details for compounds [La(C_15_H_9_O_4_)(C_12_H_8_N_2_)_2_(NO_3_)_2_]·1.5CH_3_OH (**1**), [Nd(C_15_H_9_O_4_)_2_(C_12_H_8_N_2_)_2_](NO_3_)·0.5CH_3_OH (**2**), and [Eu(C_15_H_9_O_4_)_2_(C_12_H_8_N_2_)(NO_3_)] (**3**).

Compound	1	2	3
Chemical formula	C_39_H_25_LaN_6_O_10_·1.5(CH_4_O)	C_54_H_34_N_4_NdO_8_·NO_3_·0.5(CH_4_O)	C_42_H_26_EuN_3_O_11_
*M*_r_	924.63	1089.15	900.64
Crystal system	Triclinic	Triclinic	Monoclinic
Space group	*P*ī	*P*ī	*P*2_1_/*n*
Temperature (K)	130	130	295
*a* (Å)	11.143 (7)	12.8739 (5)	13.0314 (9)
*b* (Å)	13.166 (10)	13.5942 (5)	20.2573 (14)
*c* (Å)	15.147 (13)	13.7884 (6)	15.0399 (10)
α (°)	66.648 (17)	81.598 (2)	90
β (°)	83.183 (17)	75.113 (2)	113.784 (3)
γ (°)	89.09 (2)	84.528 (2)	90
*V* (Å^3^)	2025 (3)	2302.87 (16)	3633.1 (4)
*Z*	2	2	4
Radiation type	Mo *K*α	Mo *K*α	Mo *K*α
µ (mm^−1^)	1.12	1.20	1.80
Crystal size (mm)	0.15 × 0.14 × 0.04	0.12 × 0.05 × 0.04	0.16 × 0.09 × 0.05
Diffractometer	Bruker Kappa Apex2		
Absorption correction	Numerical		
*T*_min_, *T*_max_	0.85, 0.96	0.94, 0.95	0.85, 0.91
No. of reflections			
measured	57,880	40,997	39,612
independent	7685	8826	6946
observed [*I* > 2.0σ(*I*)]	5730	6610	5373
*R*_int_	0.066	0.071	0.033
(sin θ/λ)_max_ (Å^−1^)	0.619	0.614	0.613
*R*[*F*^2^ > 2σ(*F*^2^)]	0.044	0.041	0.032
*wR*(*F*^2^)	0.062	0.074	0.053
*S*	1.00	1.00	1.00
No. of reflections	5730	6610	5373
No. of parameters	559	656	514
H-atom treatment	H-atom parameters constrained		
No. of restraints	-	3	-
Δρ_max_, Δρ_min_ (e Å^−3^)	1.51, −1.53	0.81, −0.50	0.84, −0.71

**Table 2:** Selected interatomic distances (Å) and bond angles (°) for [La(C_15_H_9_O_4_)(C_12_H_8_N_2_)_2_(NO_3_)_2_]·1.5CH_3_OH (**1**), [Nd(C_15_H_9_O_4_)_2_(C_12_H_8_N_2_)_2_](NO_3_)·0.5CH_3_OH (**2**), and [Eu(C_15_H_9_O_4_)_2_(C_12_H_8_N_2_)(NO_3_)] (**3**).

	1		2		3	
**Bond Distances (Å)**	La(1)—O(1)	2.442(3)	Nd(1)—O(1)	2.431(3)	Eu(1)—O(1)	2.343(3)
	La(1)—O(2)	2.376(3)	Nd(1)—O(2)	2.304(3)	Eu(1)—O(2)	2.315(2)
	La(1)—O(5)	2.613(4)	Nd(1)—O(5)	2.423(3)	Eu(1)—O(5)	2.316(2)
	La(1)—O(6)	2.636(3)	Nd(1)—O(6)	2.291(3)	Eu(1)—O(6)	2.295(2)
	La(1)—N(1)	2.711(4)	Nd(1)—N(1)	2.674(4)	Eu(1)—O(9)	2.492(3)
	La(1)—N(2)	2.718(4)	Nd(1)—N(2)	2.692(4)	Eu(1)—O(10)	2.529(3)
	La(1)—N(3)	2.760(4)	Nd(1)—N(3)	2.687(4)	Eu(1)—N(1)	2.596(3)
	La(1)—N(4)	2.744(4)	Nd(1)—N(4)	2.687(4)	Eu(1)—N(2)	2.604(3)
**Bond angles (°)**	O(1)—La(1)—O(2)	68.73(11)	O(1)—Nd(1)—O(2)	70.92(10)	O(1)—Eu(1)—O(2)	72.24(9)
	O(1)—La(1)—O(5)	77.45(12)	O(1)—Nd(1)—O(5)	145.45(10)	O(1)—Eu(1)—O(5)	142.04(9)
	O(2)—La(1)—O(6)	114.54(12)	O(2)—Nd(1)—O(5)	86.33(11)	O(2)—Eu(1)—O(5)	82.66(9)
	O(5)—La(1)—O(6)	48.55(10)	O(2)—Nd(1)—O(6)	95.61(12)	O(2)—Eu(1)—O(6)	106.05(9)
	O(2)—La(1)—O(8)	82.87(12)	O(5)—Nd(1)—O(6)	70.88(10)	O(1)—Eu(1)—O(9)	86.73(10)
	O(5)—La(1)—O(8)	154.41(9)	O(1)—Nd(1)—N(1)	76.05(11)	O(2)—Eu(1)—O(9)	94.78(10)
	O(6)—La(1)—O(8)	135.85(10)	O(5)—Nd(1)—N(1)	121.65(11)	O(5)—Eu(1)—O(9)	124.11(9)
	O(1)—La(1)—O(9)	119.36(11)	O(6)—Nd(1)—N(1)	79.54(11)	O(6)—Eu(1)—O(9)	154.64(9)
	O(6)—La(1)—O(9)	171.43(10)	O(1)—Nd(1)—N(2)	135.69(11)	O(1)—Eu(1)—O(10)	124.40(9)
	O(1)—La(1)—N(1)	76.21(13)	O(2)—Nd(1)—N(2)	151.34(11)	O(6)—Eu(1)—O(10)	147.92(9)
	O(2)—La(1)—N(1)	139.56(11)	O(5)—Nd(1)—N(2)	73.75(11)	O(9)—Eu(1)—O(10)	50.11(10)
	O(5)—La(1)—N(1)	116.54(11)	O(6)—Nd(1)—N(2)	96.99(12)	O(5)—Eu(1)—N(1)	73.47(10)
	O(6)—La(1)—N(1)	68.17(12)	N(1)—Nd(1)—N(2)	61.17(11)	O(6)—Eu(1)—N(1)	89.42(9)
	O(6)—La(1)—N(2)	107.33(12)	N(1)—Nd(1)—N(3)	121.61(11)	O(9)—Eu(1)—N(1)	80.43(10)
	O(2)—La(1)—N(4)	77.53(12)	N(1)—Nd(1)—N(4)	75.13(11)	O(1)—Eu(1)—N(2)	75.72(10)
	N(1)—La(1)—N(2)	61.16(13)	N(2)—Nd(1)—N(3)	75.80(11)	O(6)—Eu(1)—N(2)	80.26(9)
	N(1)—La(1)—N(3)	88.80(12)	N(2)—Nd(1)—N(4)	82.58(12)	O(10)—Eu(1)—N(2)	114.29(10)
	N(1)—La(1)—N(4)	141.88(12)	N(3)—Nd(1)—N(4)	61.20(11)	N(1)—Eu(1)—N(2)	63.76(11)

**Table 3:** Binding parameters following analysis with Stern–Volmer and Scatchard equations.

Compound	T (K)	K_SV_/10^−5^ (M^−1^)	K_q_/10^−13^ (M^−1^s^−1^)	K_b_ (M^−1^)	n
**1**	293.15	1.56 ± 0.21	1.56 ± 0.21	3.71 ± 0.23 × 10^2^	0.51 ± 0.05
	303.15	1.60 ± 0.17	1.60 ± 0.17	6.35 ± 0.10 × 10^2^	0.56 ± 0.05
	310.15	1.46 ± 0.23	1.46 ± 0.23	3.80 ± 0.44 × 10^3^	0.71 ± 0.08
**2**	293.15	5.89 ± 0.10	5.89 ± 0.10	1.50 ± 0.32 × 10^6^	1.06 ± 0.01
	303.15	4.88 ± 0.11	4.88 ± 0.11	1.40 ± 0.40 × 10^6^	1.06 ± 0.03
	310.15	4.54 ± 0.44	4.54 ± 0.44	1.33 ± 0.03 × 10^6^	1.06 ± 0.08
**3**	293.15	2.34 ± 0.14	2.34 ± 0.14	2.04 ± 0.10 × 10^4^	0.81 ± 0.10
	303.15	2.76 ± 0.26	2.76 ± 0.26	2.10 ± 0.37 × 10^5^	0.98 ± 0.05
	310.15	2.06 ± 0.31	2.06 ± 0.31	4.80 ± 0.64 × 10^6^	1.25 ± 0.09

**Table 4:** Thermodynamic data of **1**–**3** from Vant Hoff’s equation.

Compound	T (K)	ΔH (kJ/mol)	ΔS (kJ/molK)	ΔG (kJ/mol)
**1**	293.15	99.26 ± 17.38	0.386 ± 0.16	−13.90 ± 1.54
	303.15			−17.76 ± 2.30
	310.15			−20.46 ± 1.85
**2**	293.15	−7.39 ± 1.96	0.093 ± 0.04	−34.65 ± 0.96
	303.15			−35.58 ± 5.40
	310.15			−36.23 ± 0.58
**3**	293.15	237.31 ± 5.90	0.89 ± 0.01	−23.59 ± 2.79
	303.15			−32.49 ± 1.25
	310.15			−38.72 ± 2.90

**Table 5:** BSA α-helical content (%) as a function of BSA–metal compound molar ratio upon addition of **1** and **3**.

α-Helical Content (%)		
BSA–Metal Compound Molar Ratio	1	3
1:0	51.38	51.38
1:1	47.43	37.56
1:2	51.40	27.68

## Data Availability

Data are contained within the article and Supplementary Materials.

## Supplementary Materials

The following supporting information can be downloaded at: https://www.mdpi.com/article/10.3390/ijms26031198/s1.

## Abbreviations

DMSO	Dimethyl sulfoxide
Chrysin	Chr
1,10-phenanthroline	phen
Lanthanides	Ln
BVS	Bond valence sum
ESI-MS	Electron spray ionization mass spectrometry
FT-IR	Fourier transform infrared
UV-Visible	Ultraviolet spectroscopy
VESTA	Visualization for Electronic and Structural Analysis
Trp	Tryptophan
Tyr	Tyrosine
Phe	Phenylalanine
ZOI	Zone of inhibition
MIC	Minimum inhibitory concentration
