# Supplementary material for: Hybrid Lanthanide Metal–Organic Compounds with Flavonoids: Magneto-Optical Properties and Biological Activity Profiles
Source: Int J Mol Sci. 2025 Jan 30;26(3):1198. doi: 10.3390/ijms26031198 (PMC11818910; doi:10.3390/ijms26031198)
Supplement: Supplementary file 1 [file ijms-26-01198-s001.zip › ijms-3403384-supplementary.pdf]

Supplementary Information

Hybrid lanthanide metal organic compounds with flavonoids. Magneto-optical properties and biological activity profiles.

Sevasti Matsia[1], Anastasios Papadopoulos[1], Antonios Hatzidimitriou[2], Lars Schumacher[3], Aylin Koldemir[3], Rainer Pöttgen[3], Angeliki Panagiotopoulou[4], Christos T. Chasapis[5], and Athanasios Salifoglou[1,*]

[1] Laboratory of Inorganic Chemistry and Advanced Materials, School of Chemical Engineering, Aristotle University of Thessaloniki, Thessaloniki 54124, Greece
[2] Laboratory of Inorganic Chemistry, Department of Chemistry, Aristotle University of Thessaloniki, Thessaloniki 54124, Greece
[3] Institut für Anorganische und Analytische Chemie, Universität Münster, Corrensstrasse 30, D-48149 Münster, Germany
[4] Institute of Biosciences & Applications, NCSR "Demokritos", Aghia Paraskevi, Attiki 15310, Greece
[5] Institute of Chemical Biology, National Hellenic Research Foundation, Athens 11635, Greece

* Correspondence: salif@auth.gr; Tel.:+30-2310-996-179

Tables

Table S1. Hydrogen-bond geometry in compounds **1**, **2** and **3**.

D—H···*A*	*D*—H (Å)	H···*A* (Å)	*D*···*A* (Å)	*D*—H···*A* (º)	Symmetry code*
**1**					
O4—H111···O9[i]	0.82	2.47	3.003 (8)	124	(i) −*x*+1, −*y*+2, −*z*+1
O4—H111···O10[i]	0.82	2.09	2.908 (8)	180	(i) −*x*+1, −*y*+2, −*z*+1
O11—H112···O12	0.82	2.31	3.078 (8)	155	
O12—H122···O5	0.83	2.22	3.031 (8)	165	
O12—H122···O7	0.83	2.39	3.046 (8)	137	
O12—H122···N5	0.83	2.58	3.384 (8)	164	
O13—H131···O11[ii]	0.83	2.29	3.066 (8)	157	(ii) −*x*+2, −*y*+1, −*z*+2
**2**					
O4—H42···O10[i]	0.83	1.94	2.754 (9)	167	(i) *x*−1, *y*+1, *z*
O4—H42···O11[i]	0.83	2.47	3.098 (9)	133	(i) *x*−1, *y*+1, *z*
O4—H42···N5[i]	0.83	2.54	3.329 (9)	159	(i) *x*−1, *y*+1, *z*
O8—H81···O9[ii]	0.82	1.90	2.675 (9)	158	(ii) −*x*+1, −*y*+1, −*z*+1
O8—H81···N5[ii]	0.82	2.57	3.163 (9)	131	(ii) −*x*+1, −*y*+1, −*z*+1
**3**					
O4—H42···O6[i]	0.81	1.91	2.722 (7)	177	(i) −*x*+1/2, *y*−1/2, −*z*+1/2
O8—H81···O2[ii]	0.81	1.91	2.710 (7)	168	(ii) −*x*, −*y*+1, −*z*

* (ii) and (ii) reflect symmetry codes characteristic of the crystallographic processes inherent to the hydrogen-bond geometry of each entry in the Table.

Table S2. MIC and ZOI values of controls and compounds **1-3**

**Concentration (mg)** ZOI (mm)	***E. coli***		***S. aureus***	
**Compound 1**	**0.05 mg**		**1.0 mg**	**10 mg**
	20.0±0.6		n.e.z.	15.5±0.2
$La(NO_3)_3$	**0.02 mg**		**0.04 mg**	**3.5 mg**
	n.e.z.		n.e.z.	n.e.z.
Chr	**0.01 mg**		**0.03 mg**	**2.7 mg**
	n.e.z.		n.e.z.	n.e.z.
phen	**0.05 mg**		**0.04 mg**	**3.9 mg**
	20.5±0.3		n.e.z.	>30.0
**Compound 2**	**0.50 mg**	**1.0 mg**	**1.0 mg**	**10 mg**
	n.e.z.	28.5±0.5	n.e.z.	16.5±0.3
$Nd(NO_3)_3•6H_2O$	**0.20 mg**	**0.40 mg**	**0.4 mg**	**4.0 mg**
	n.e.z.	n.e.z.	n.e.z.	n.e.z.
Chr	**0.23 mg**	**0.46 mg**	**0.5 mg**	**4.6 mg**
	n.e.z.	n.e.z.	n.e.z.	n.e.z.
phen	**0.16 mg**	**0.33 mg**	**0.3 mg**	**3.3 mg**
	20.5±0.7	>35.0	n.e.z.	>30.0
**Compound 3**	**1.0 mg**	**10 mg**	**1.0 mg**	**10 mg**
	n.e.z.	22.8±0.4	n.e.z.	20.5±0.2
$Eu(NO_3)_3•xH_2O$	**0.25 mg**	**5.0 mg**	**0.5 mg**	**4.9 mg**
	n.e.z.	n.e.z.	n.e.z.	n.e.z.
Chr	**0.56 mg**	**5.6 mg**	**0.6 mg**	**5.6 mg**
	n.e.z.	29.0±0.3	n.e.z.	n.e.z.
phen	**0.20 mg**	**2.0 mg**	**0.2 mg**	**2.0 mg**
	>35.0	>35.0	n.e.z.	27.0±0.1

Note: n.e.z: no evaluable zone.
1% Penicillin/Streptomycin in *E. coli* exhibits a ZOI of 19.8±0.2 mm
0.05% Penicillin/Streptomycin in *S. aureus* exhibits a ZOI of 28.8±0.5 mm

Synthesis of Europium(III) nitrate, $Eu(NO_3)_3{\cdot}xH_2O$

In a round-bottom flask, 1.81 g (5.14 mmol) of Eu_2O_3 was introduced in 15 mL of water, under continuous stirring. Subsequently, approximately 1.80 mL (25.8 mmol) of HNO_3 was added, until the solution was clear. Thereafter, the solvent was evaporated, using a rotary evaporator with a water bath temperature of 70 °C for 1.5 h. White microcrystalline material precipitated and was immediately stored in a desiccator for further use. Yield: 4.03 g (87.8%).

$$Eu_2O_3 + 3HNO_3 \xrightarrow{H_2O} 2Eu(NO_3)_3{\cdot}xH_2O$$

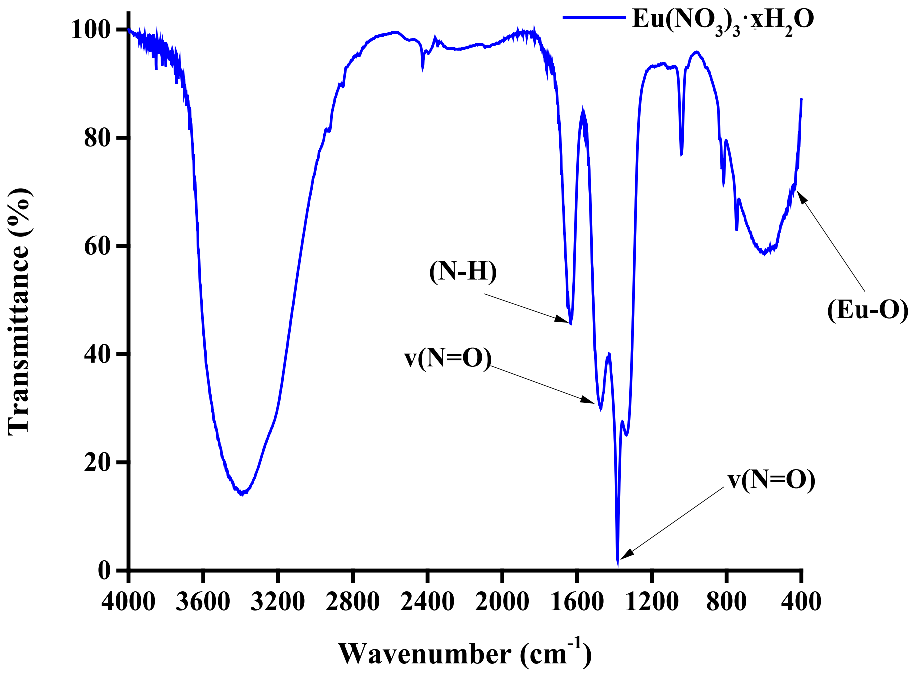

Figure S1. Synthesis and FT-IR spectrum of europium(III) nitrate.

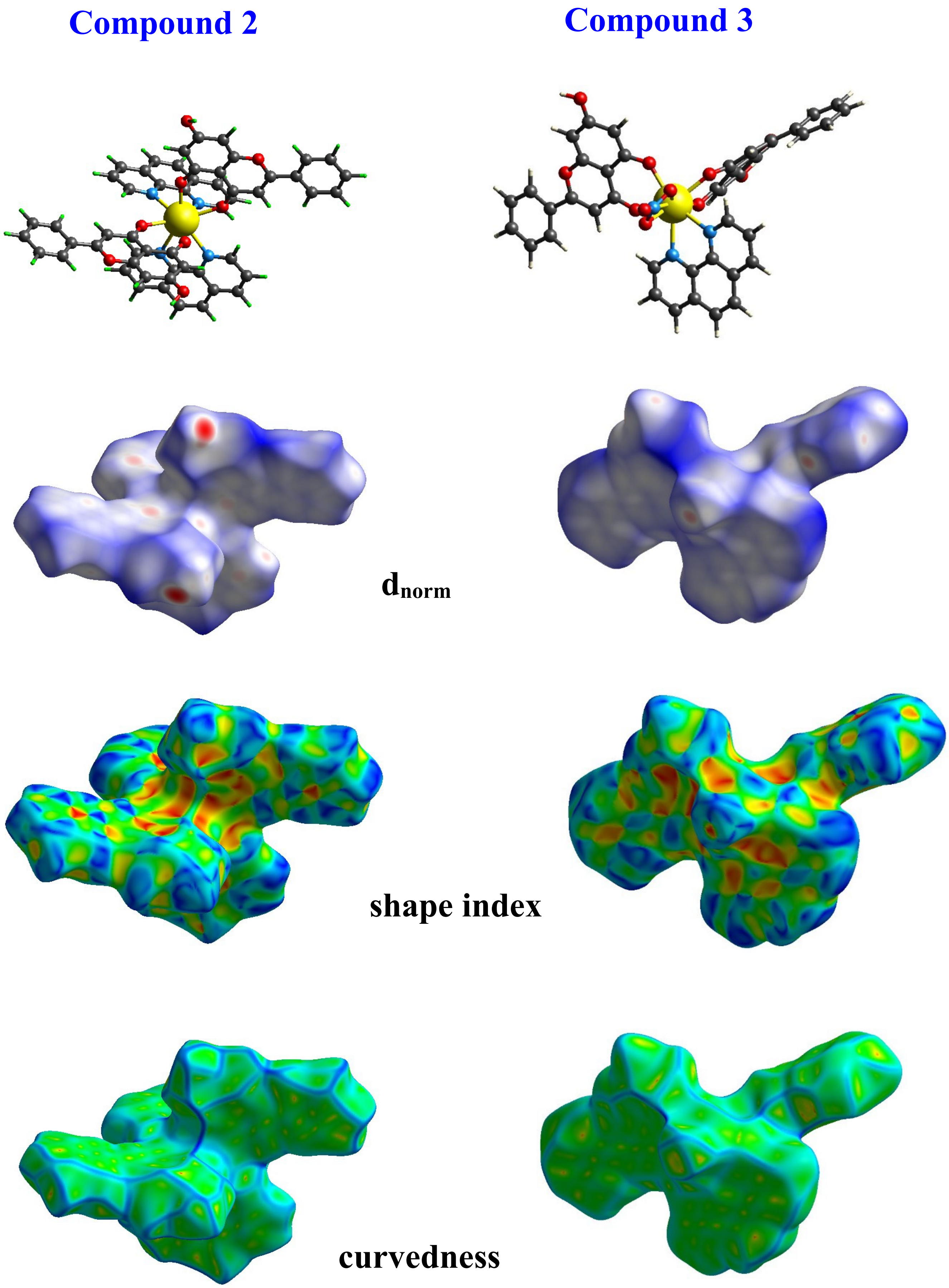

Figure S2. Hirshfeld surface analysis mapping of **2-3**.

Compound 2 **Compound 3**

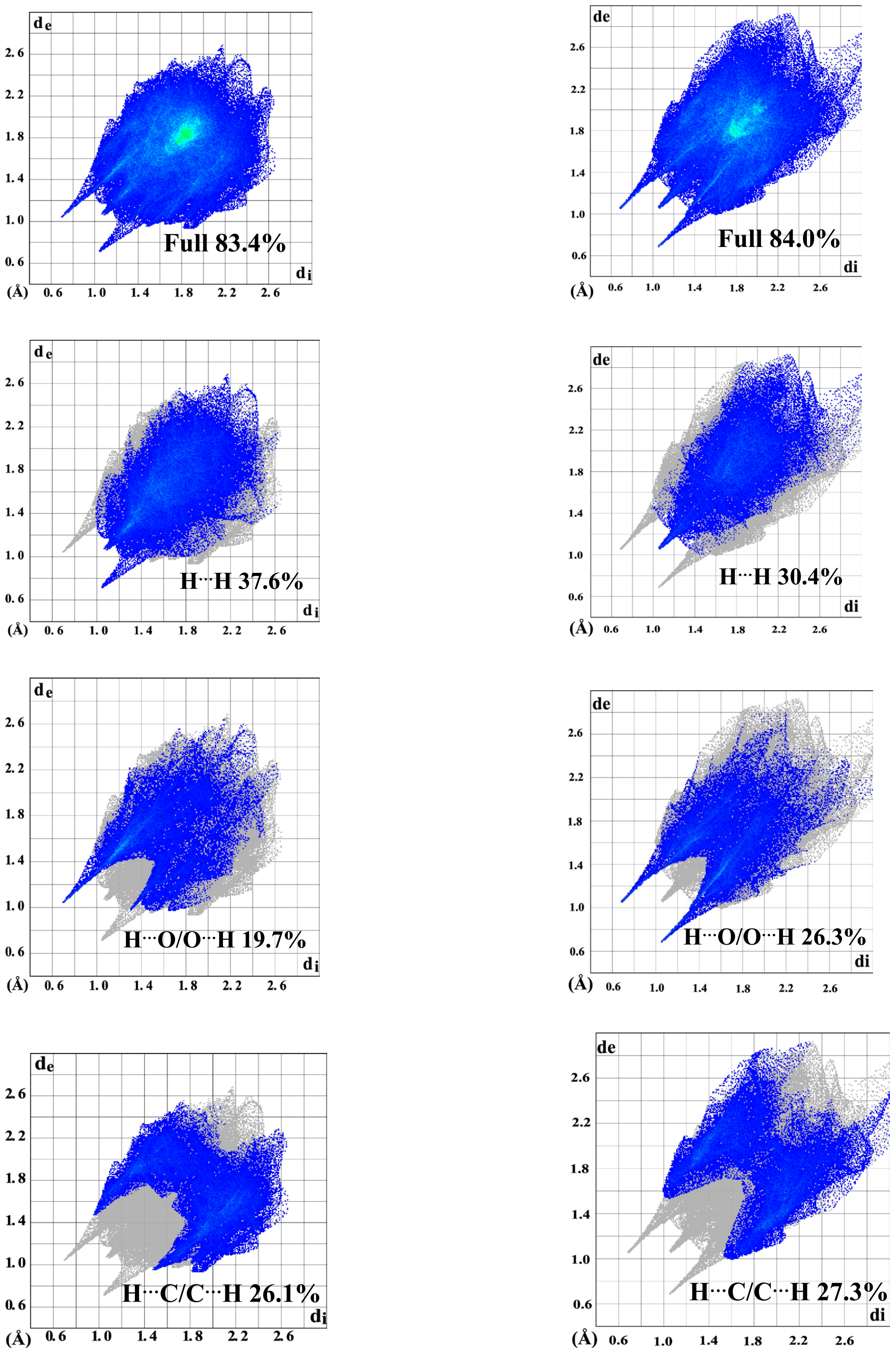

Figure S3. 2D Fingerprint Plot with relative percentage contributions of different interatomic contacts expressed over d_{norm} mapping for **2-3**.

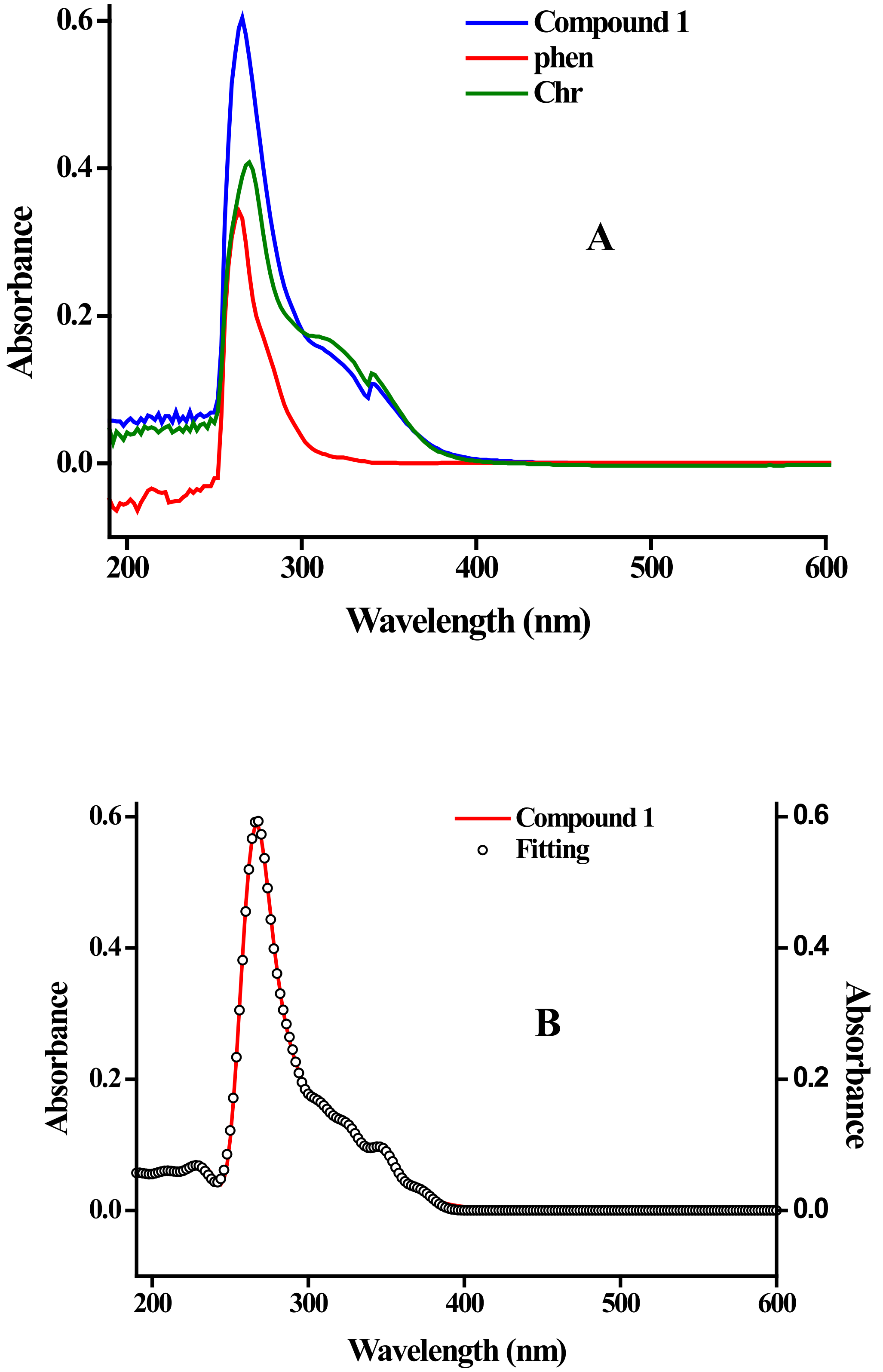

Figure S4. **A.** Comparative UV-Visible spectra of **1** with phen and Chr in DMSO at 10^{-5} M. **B.** Electronic spectrum (red line) and spectral fitting (scatter) of compound **1** in DMSO (10^{-5} M).

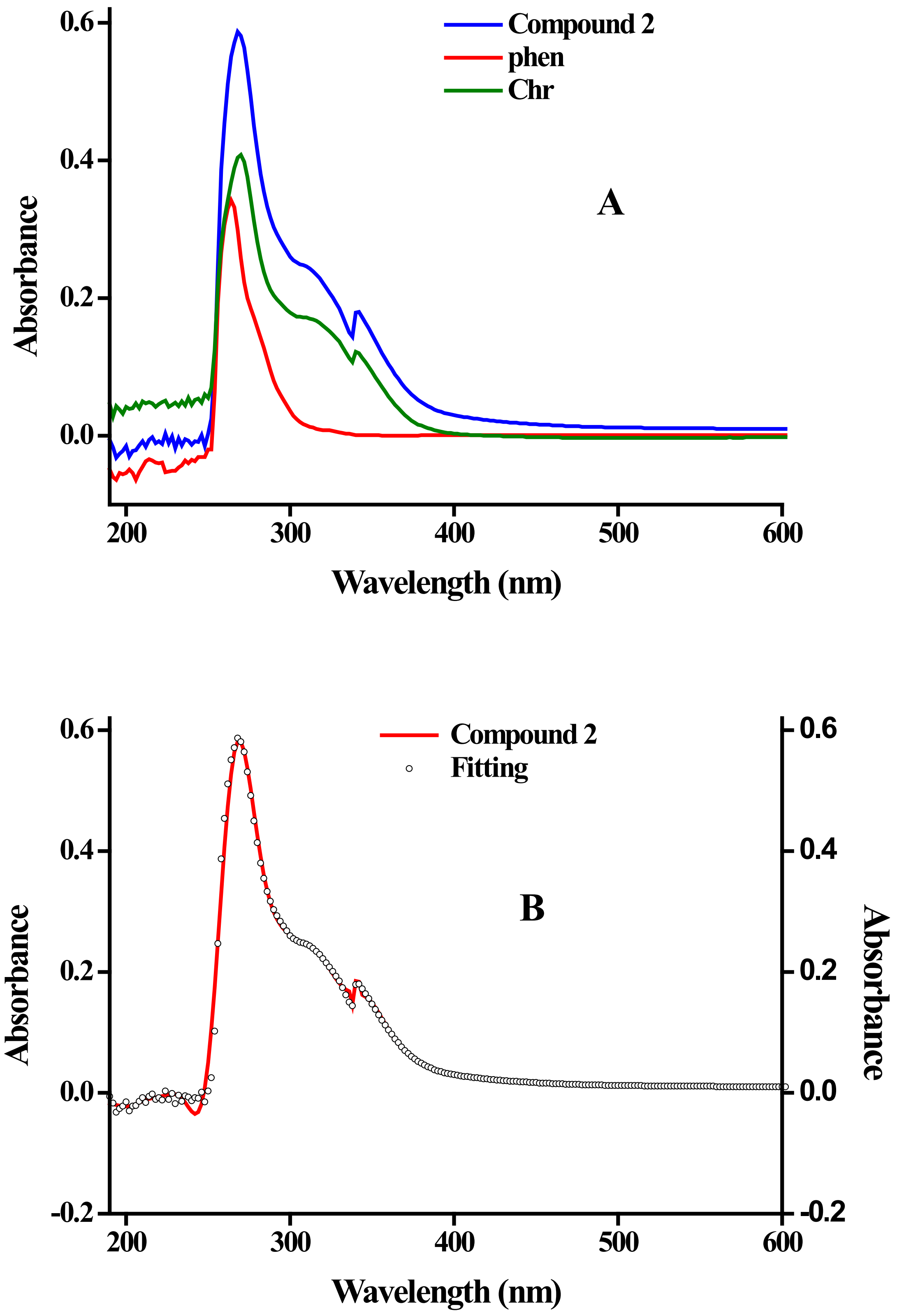

Figure S5. **A.** Comparative UV-Visible spectra of **2** with phen and Chr in DMSO at 10^{-5} M. **B.** Electronic spectrum (red line) and spectral fitting (scatter) of compound **2** in DMSO (10^{-5} M).

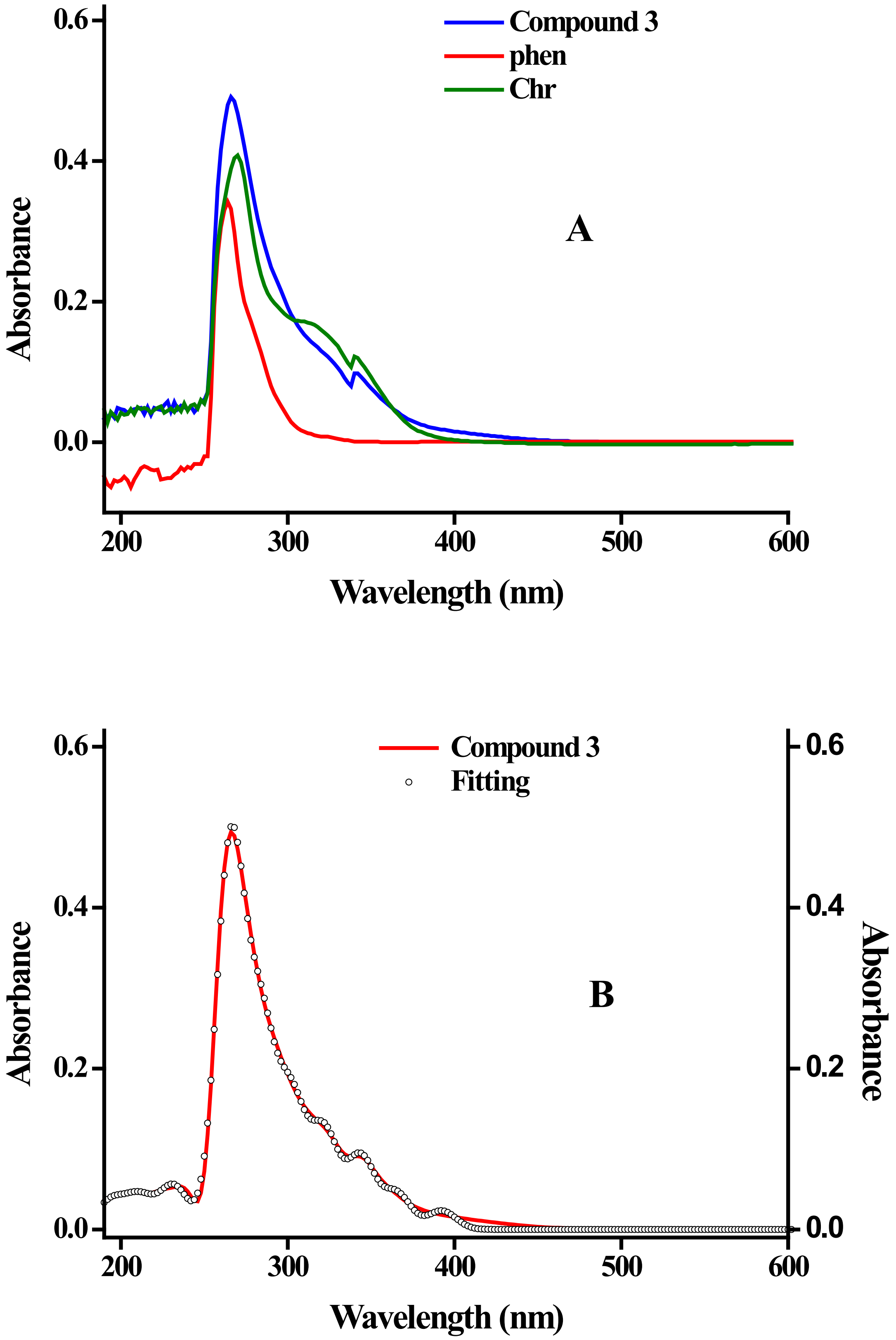

Figure S6. **A.** Comparative UV-Visible spectra of **3** with phen and Chr in DMSO at 10^{-5} M. **B.** Electronic spectrum (red line) and spectral fitting (scatter) of compound **3** in DMSO (10^{-5} M).

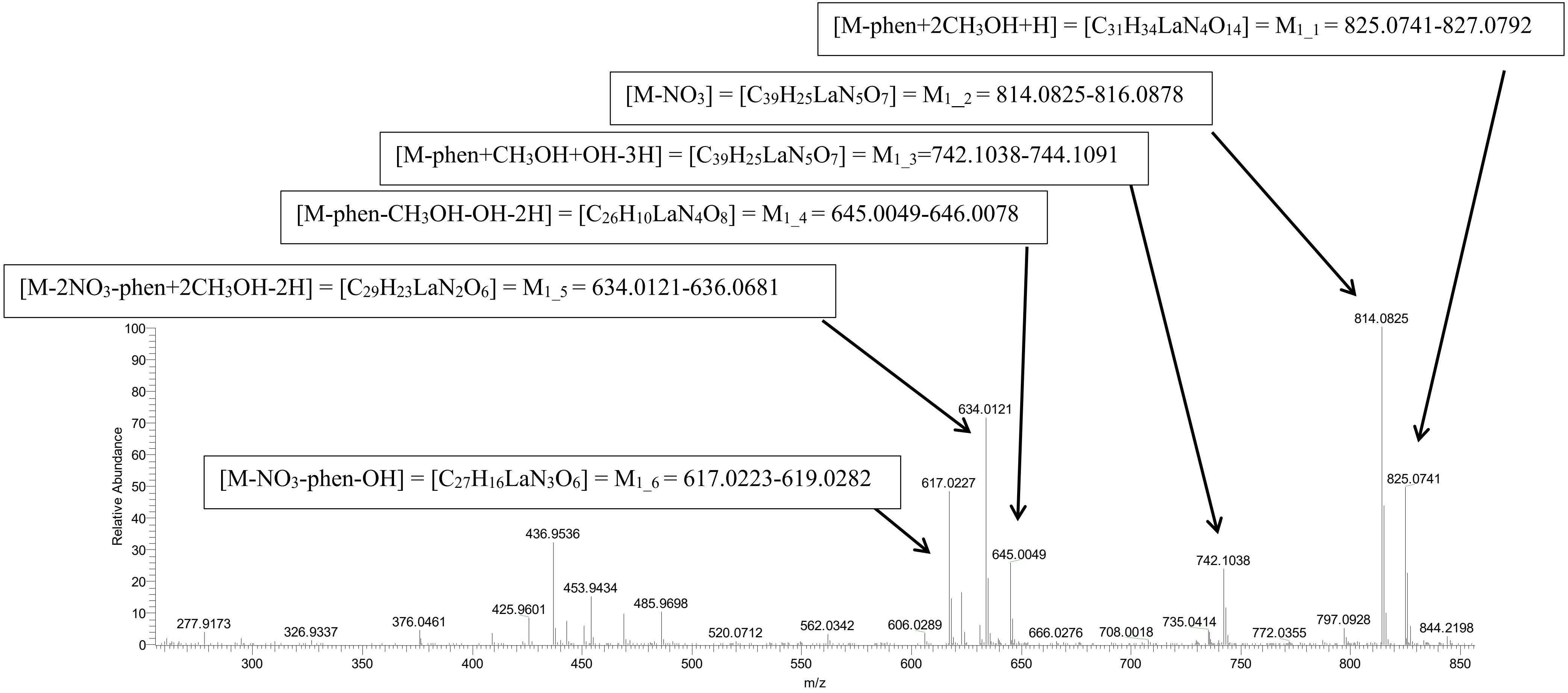

Figure S7. ESI-MS spectra of **1** and the appropriate species in methanol solution through positive mode of ionization.

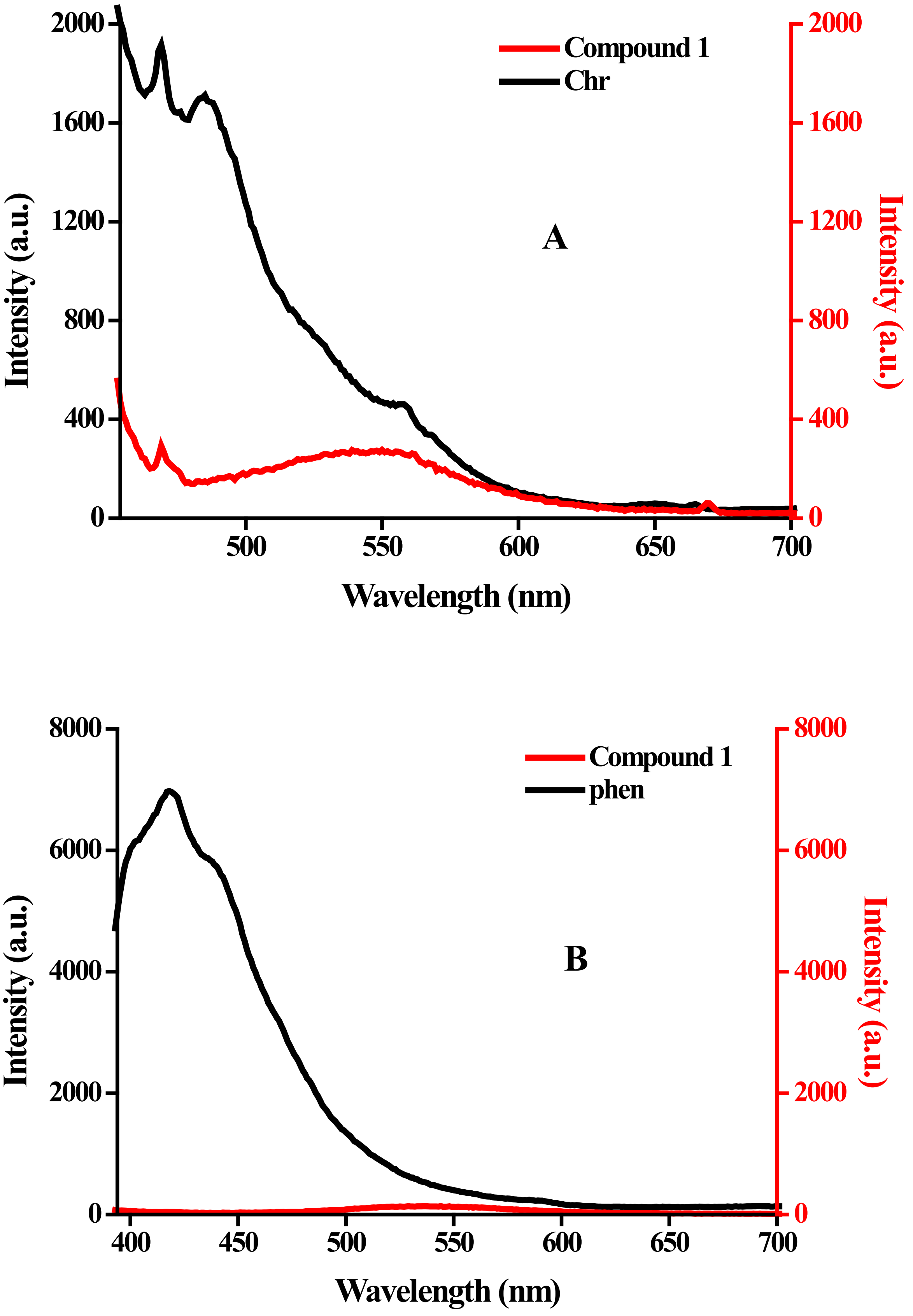

Figure S8. Comparative and normalized solid-state luminescence spectra between **1** and **A)** Chr at λ_{ex} 443 nm; **B)** phen at λ_{ex} 379 nm.

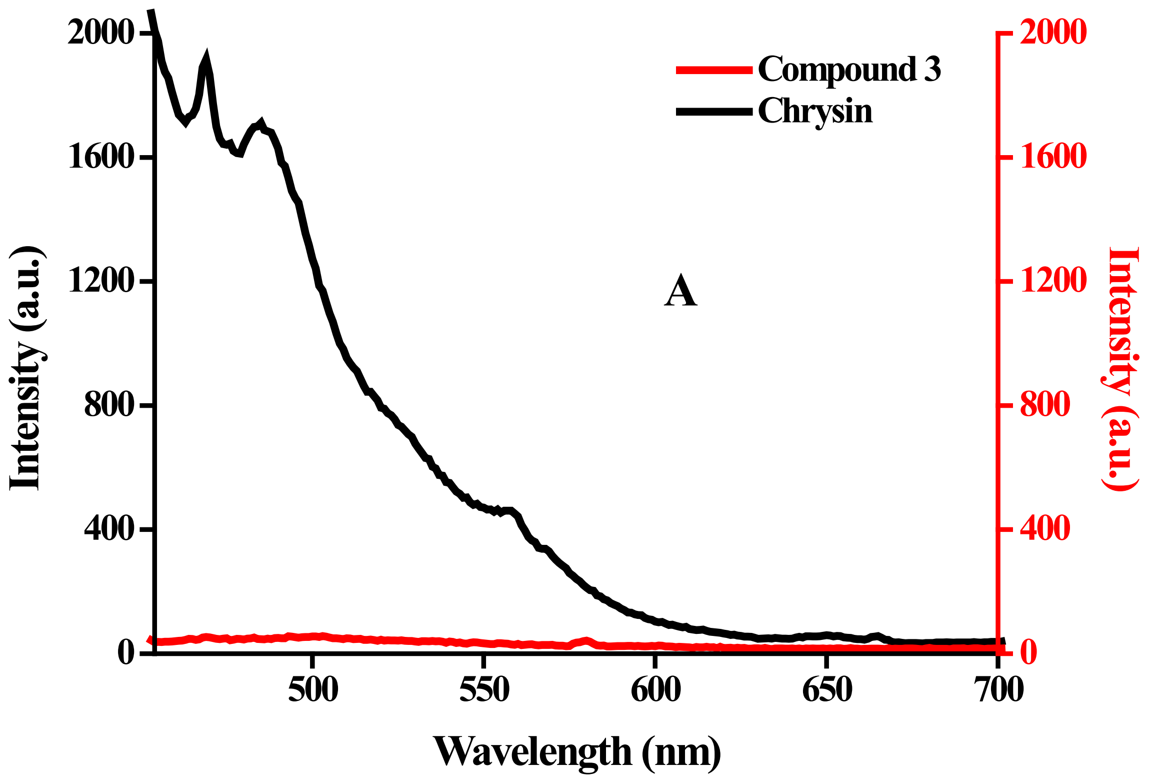

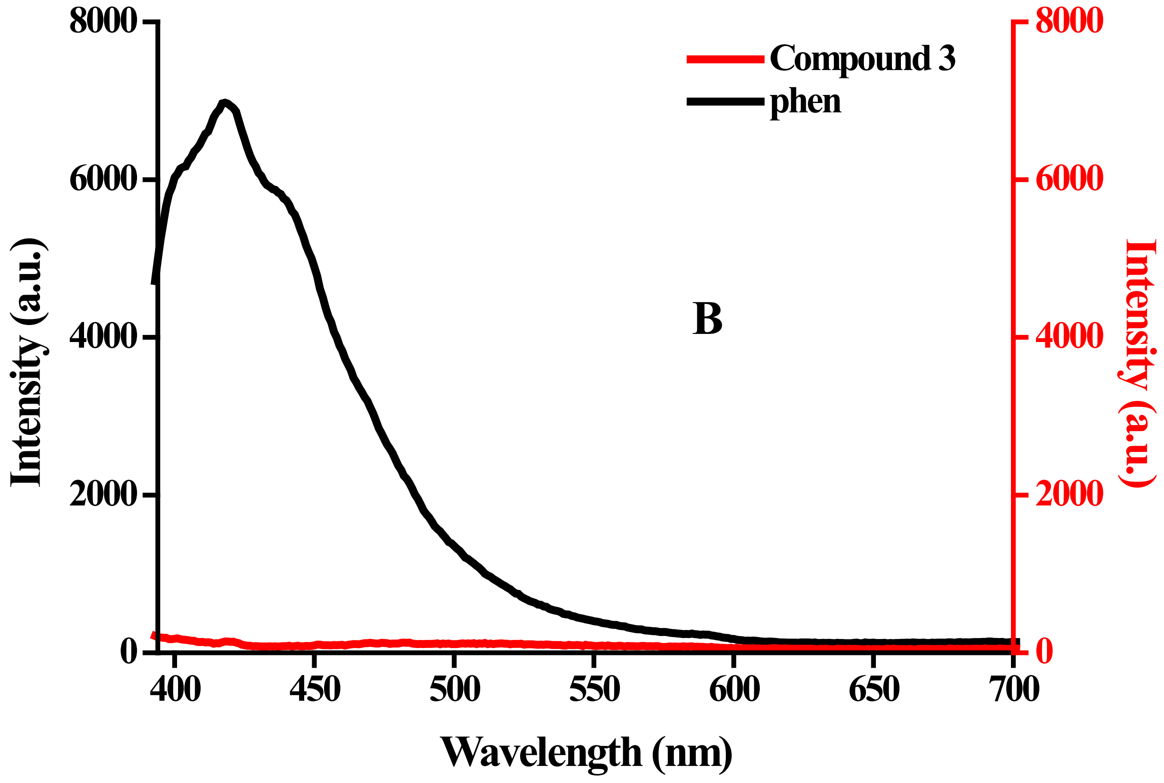

Figure S9. Comparative and normalized solid-state luminescence spectra between **3** and **A)** Chr at λ_{ex} 385 nm; **B)** phen at λ_{ex} 364 nm.

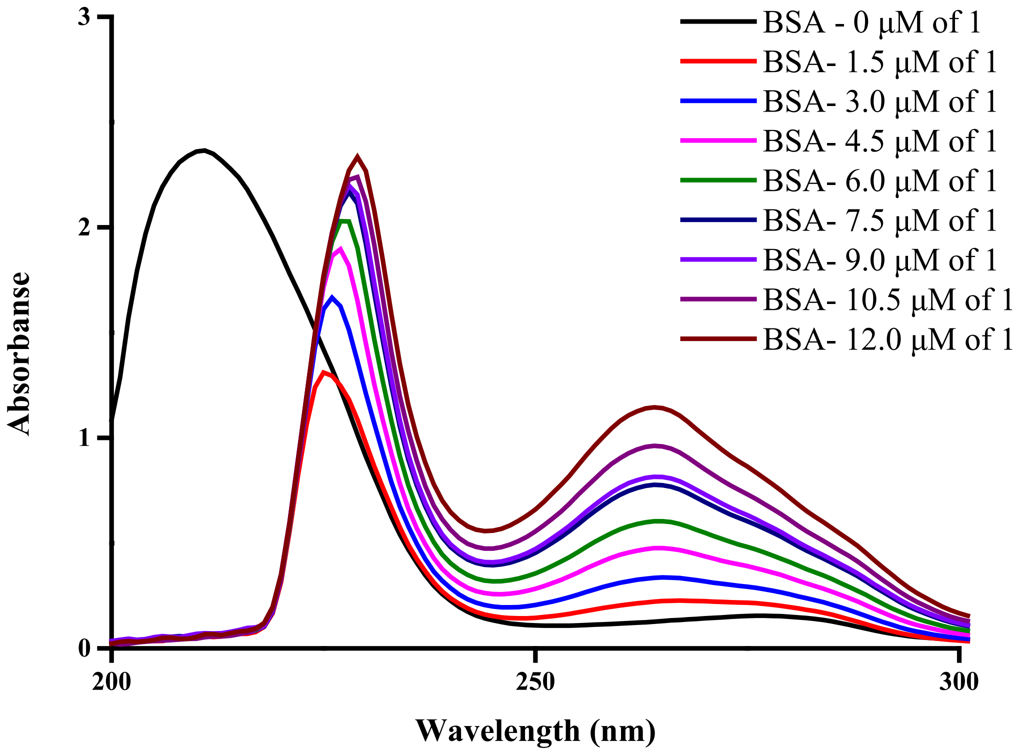

Figure S10. UV-Visible absorption spectra of solutions containing BSA (3 μM, PBS) and increasing molar ratios involving **1** (DMSO).

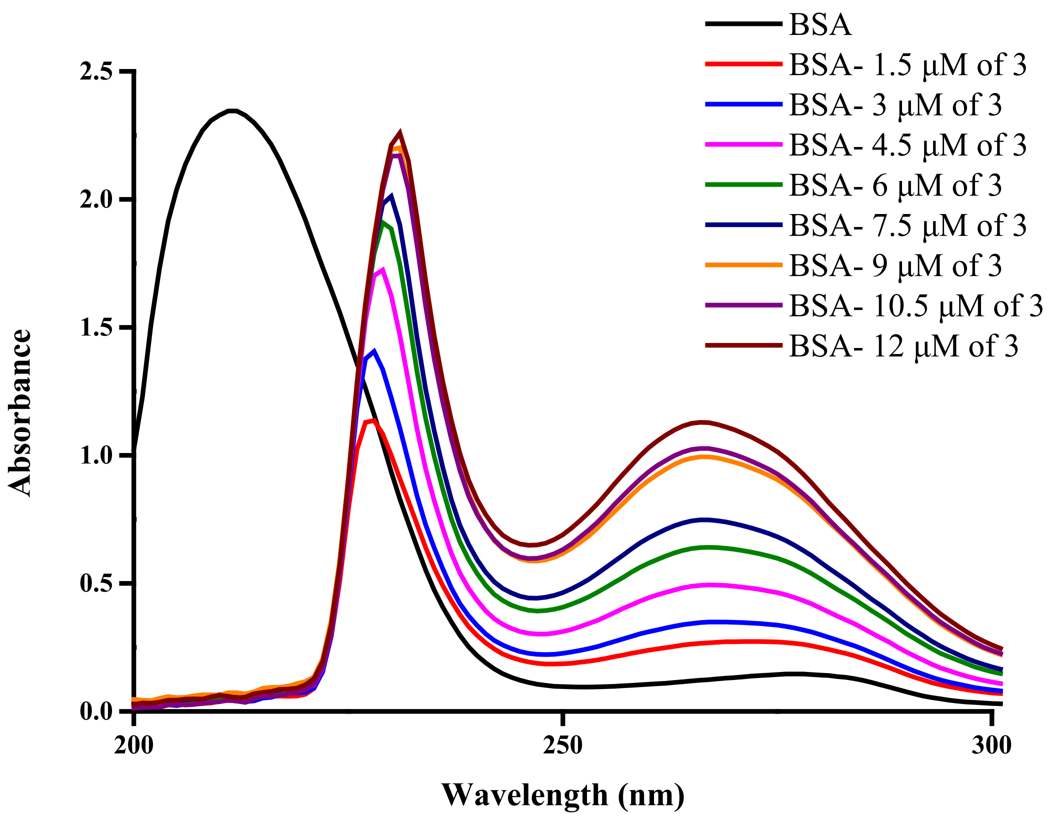

Figure S11. UV-Visible absorption spectra of solutions containing BSA (3 µM, PBS) and different molar ratios involving **3** (DMSO).

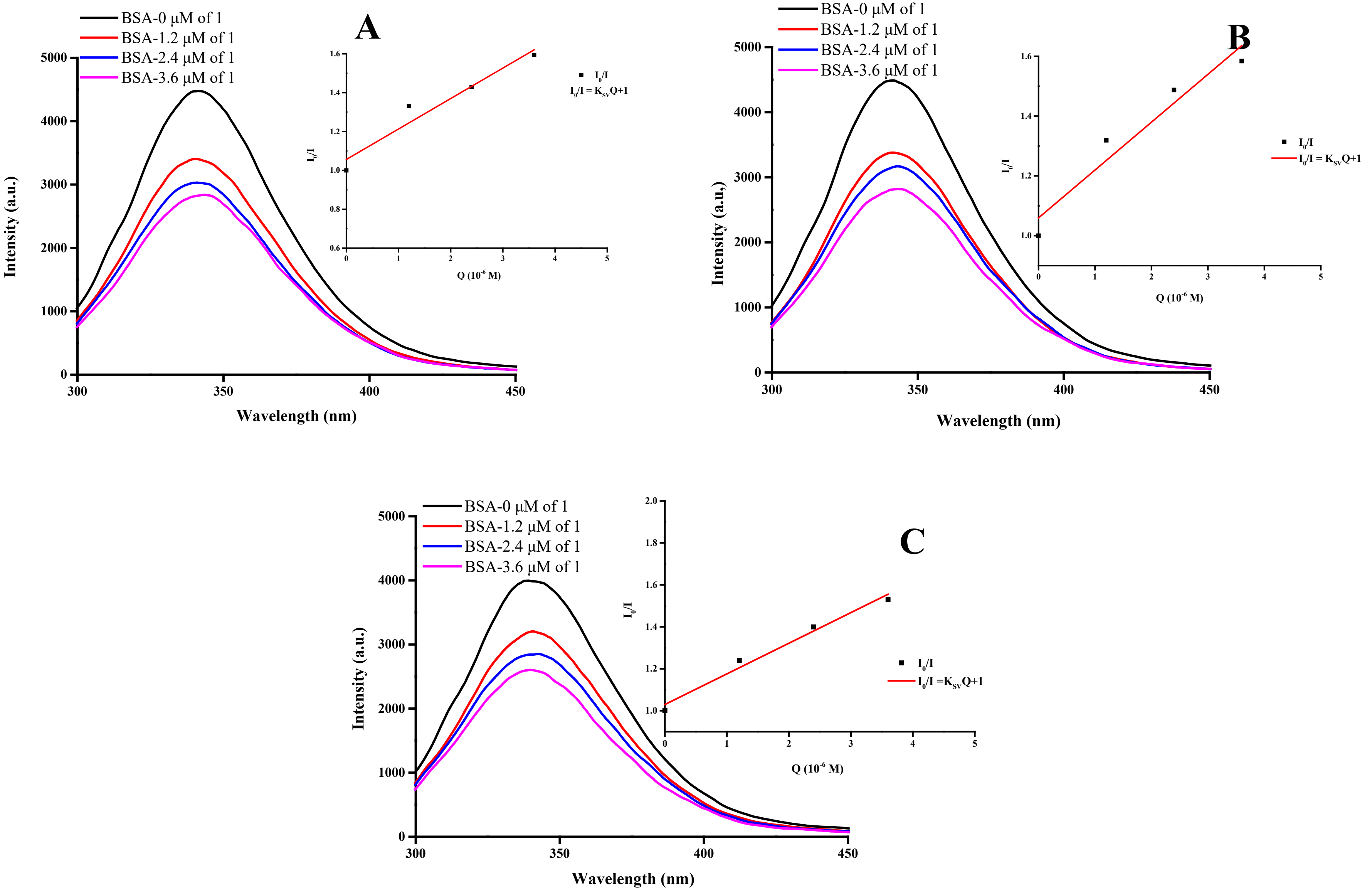

Figure S12. Fluorescence spectra of solutions containing BSA (1.5 μM, PBS) and different molar ratios involving **1** (DMSO). **Inset**: Stern-Volmer plot acquired from steady-state fluorescence at **A**) 20 °C, **B**) 30 °C, and **C**) 37 °C

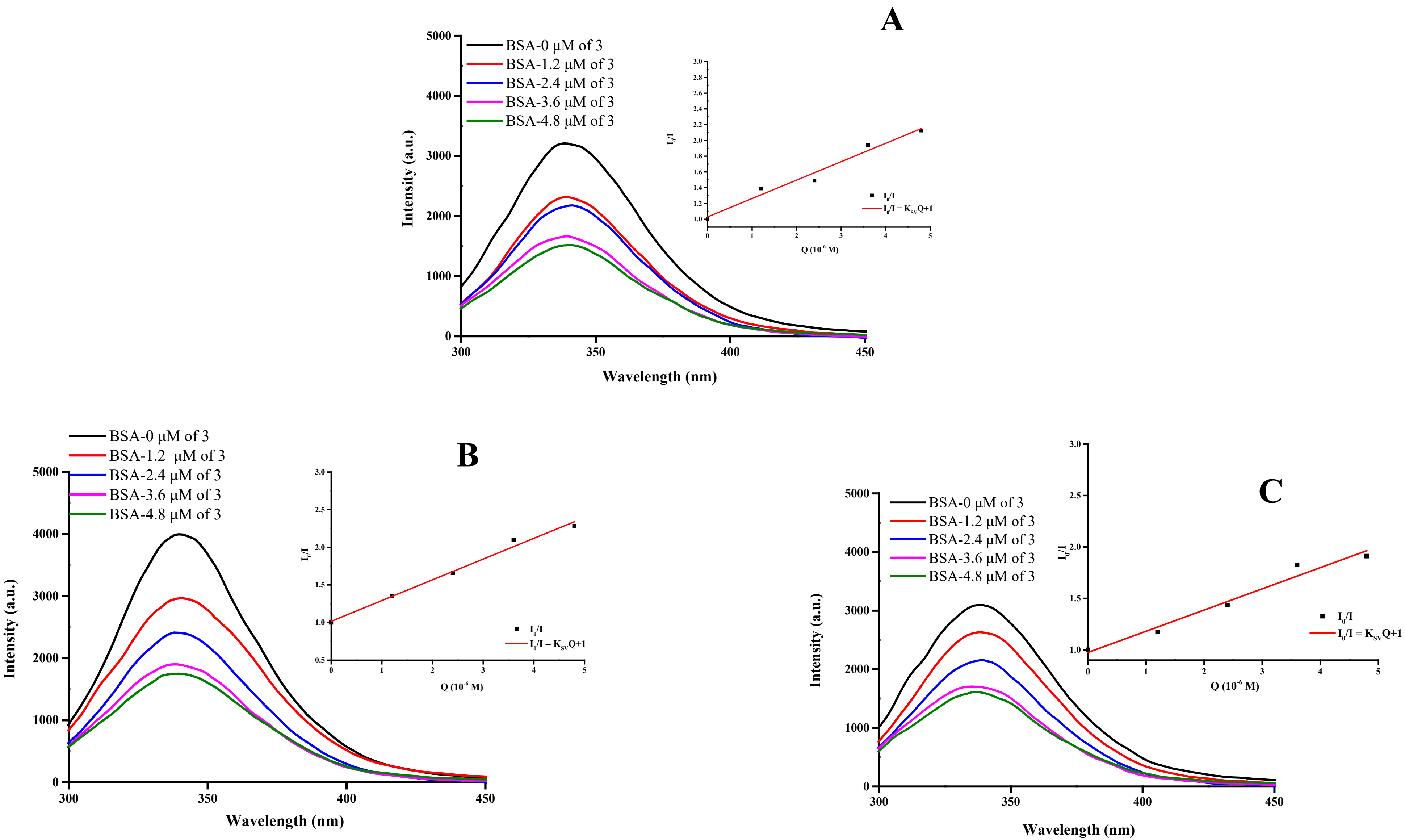

Figure S13. Fluorescence spectra of solution containing BSA (1.5 μM, PBS) and molar ratios involving **3** (DMSO). **Inset**: Stern-Volmer plot acquired from steady-state fluorescence at **A**) 20 °C, **B**) 30 °C, and **C**) 37 °C

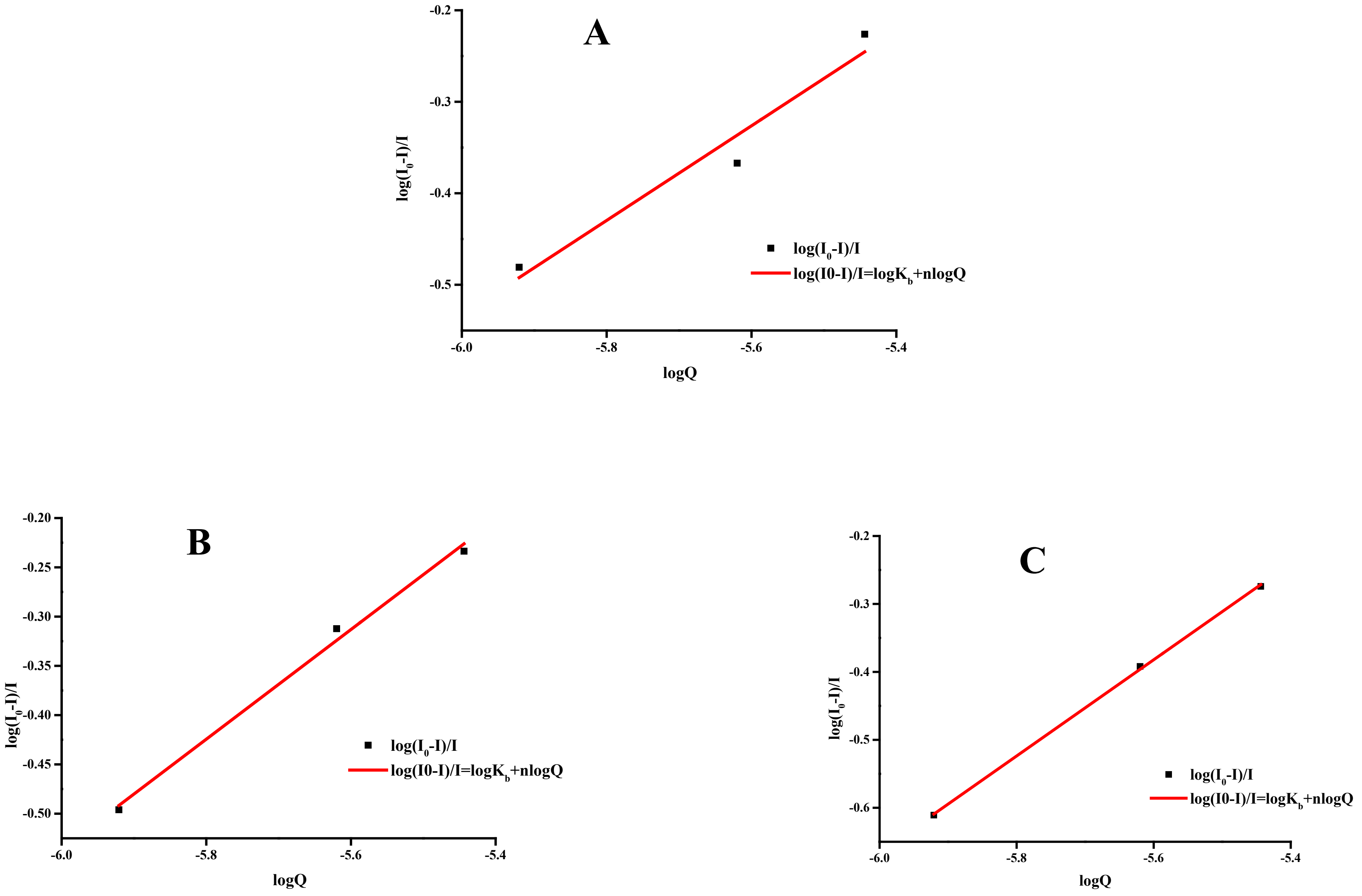

Figure S14. Scatchard plot acquired from steady-state fluorescence of La-Chr-phen at **A**) 20 °C, **B**) 30 °C, and **C**) 37 °C

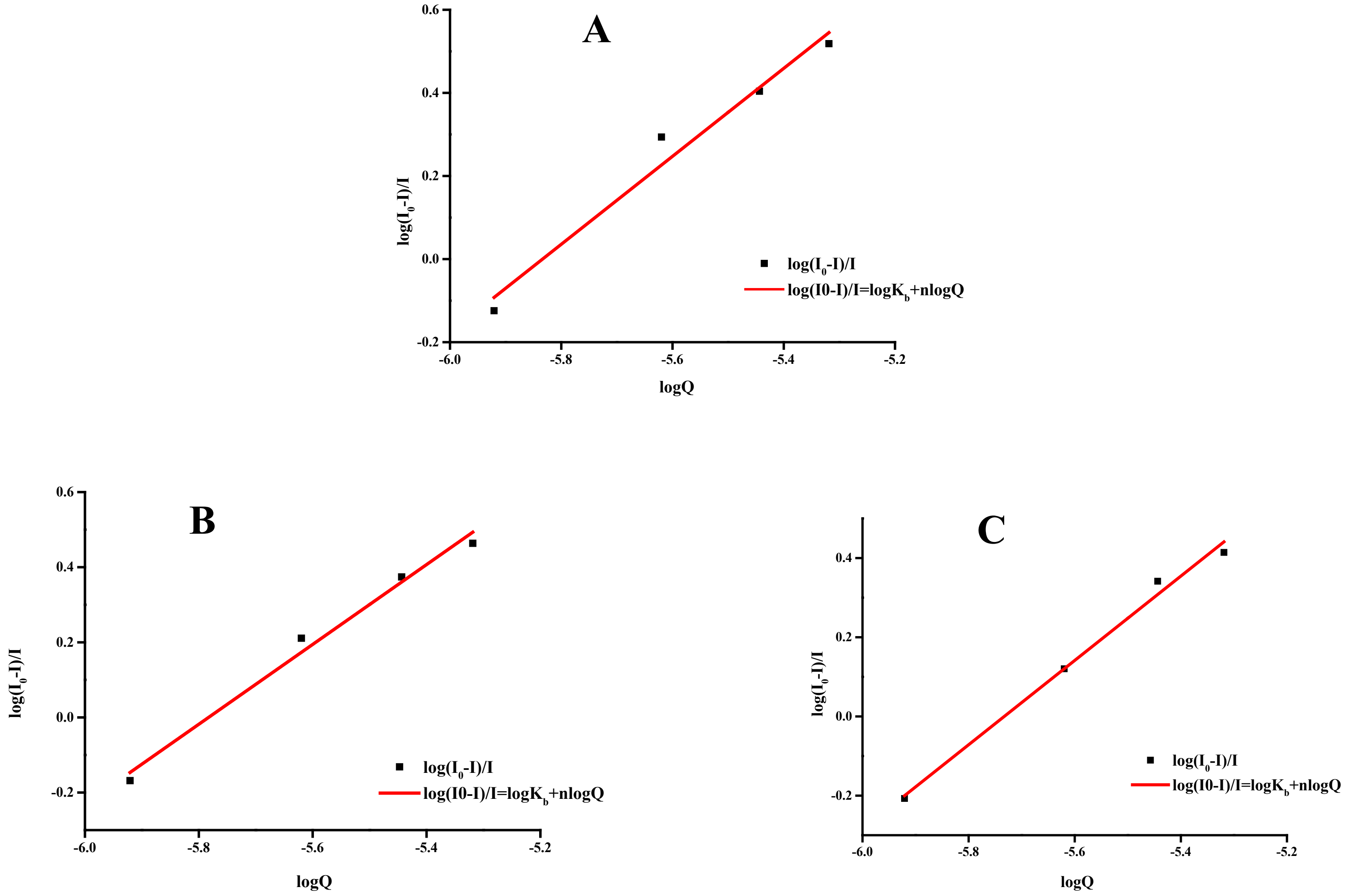

Figure S15. Scatchard plot acquired from steady-state fluorescence of Nd-Chr-phen at **A**) 20 °C, **B**) 30 °C, and **C**)

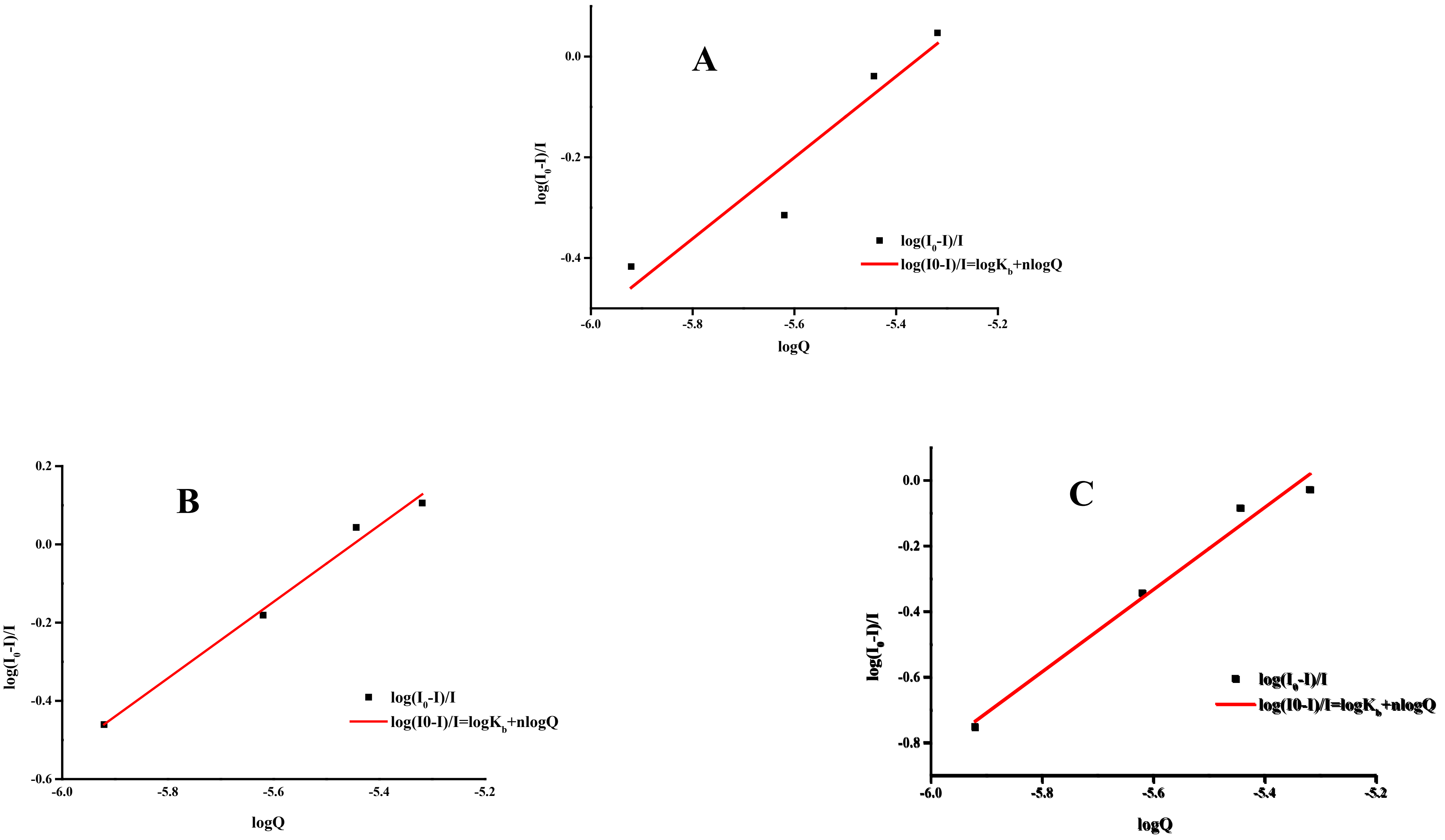

Figure S16. Scatchard plot acquired from steady-state fluorescence of Eu-Chr-phen at **A**) 20°C, **B**) 30°C, and **C**) 37°C

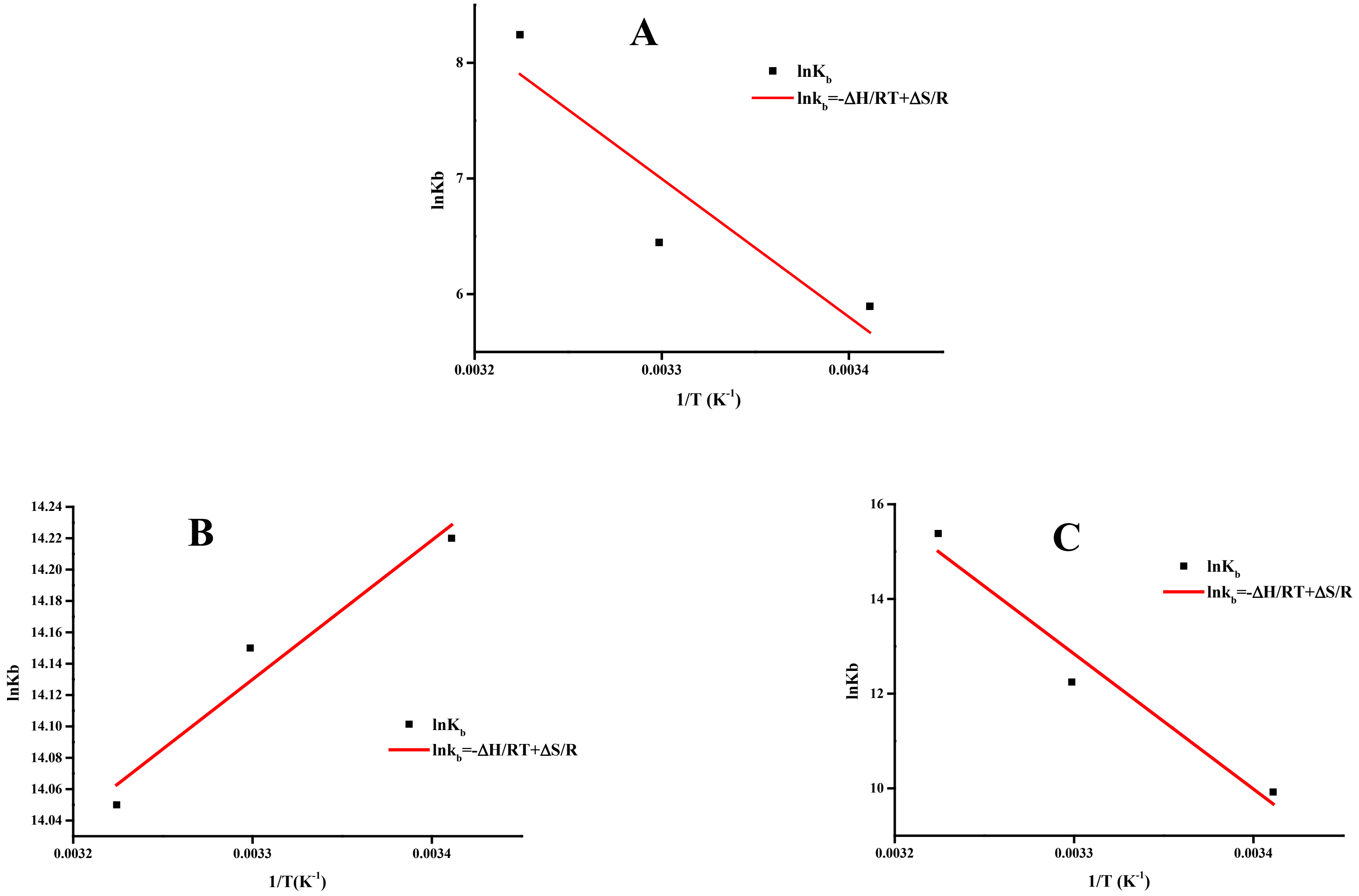

Figure S17. Van't Hoff plot acquired from steady-state fluorescence of **A**) La-Chr-phen, **B**) Nd-Chr-phen, and **C**) Eu-Chr-phen

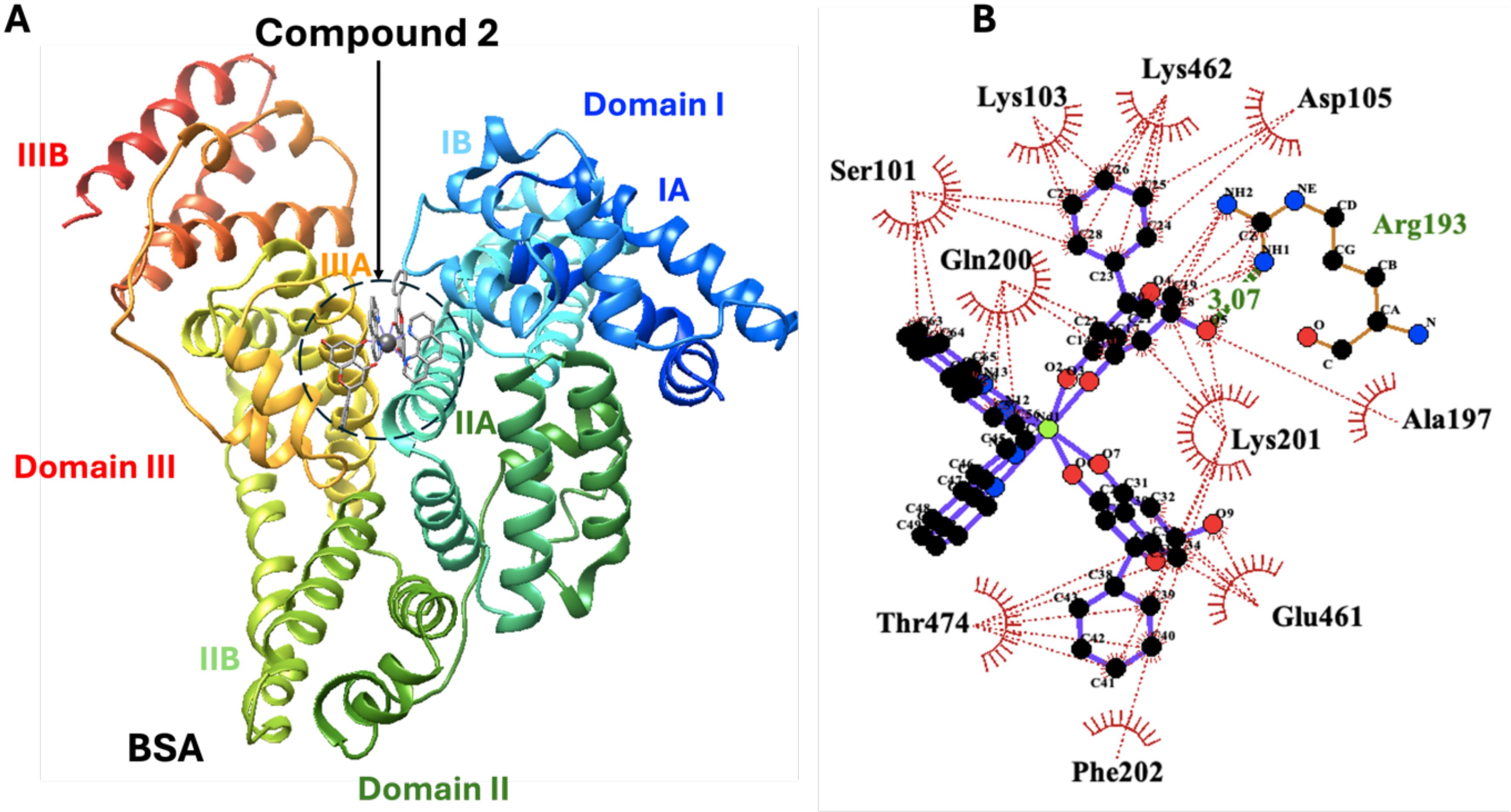

Figure S18. A. Compound **2** was docked against the 3D structure of BSA; **B.** 2D interaction diagrams illustrate the interactions between compound **2** and the BSA binding motif (hydrophobic contacts are indicated in red, and hydrogen bonds are shown in green, with bond lengths specified).

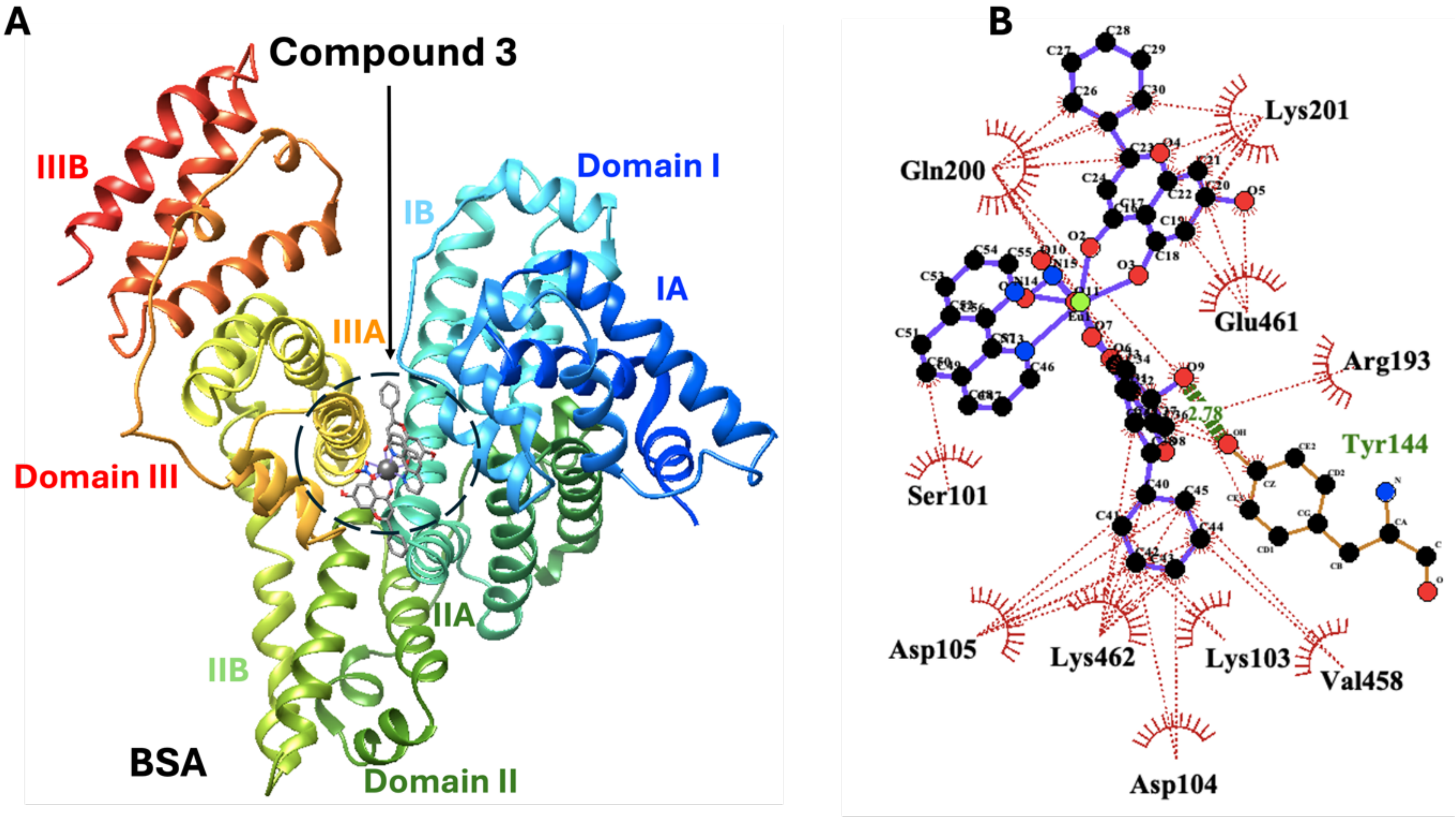

Figure S19. A. Compound **3** was docked against the 3D structure of BSA; **B.** 2D interaction diagrams illustrate the interactions between compound **3** and the BSA binding motif (hydrophobic contacts are indicated in red, and hydrogen bonds are shown in green, with bond lengths specified).

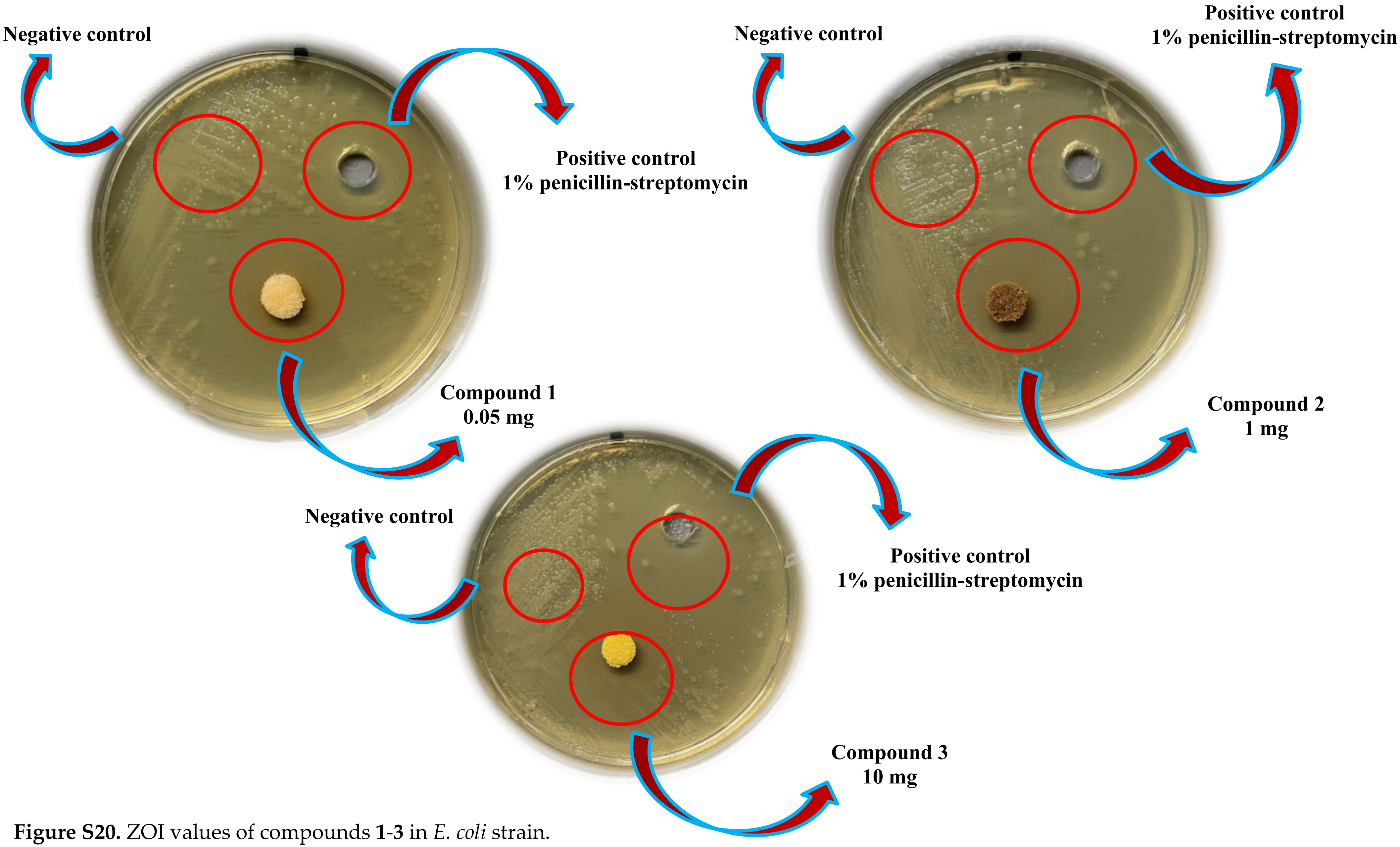

Figure S20. ZOI values of compounds **1-3** in *E. coli* strain.

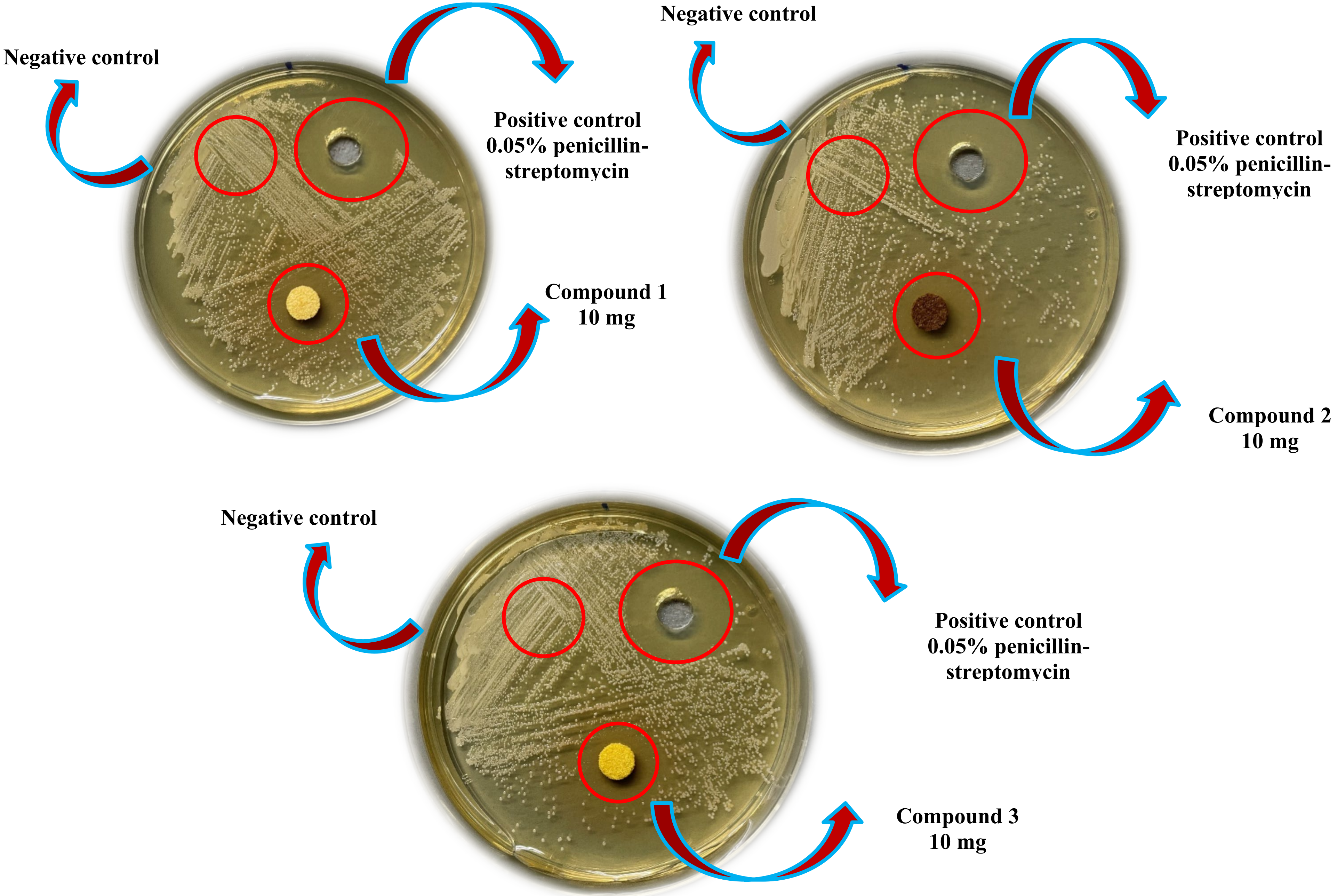

Figure S21. ZOI of compounds **1-3** in *S. aureus* following incubation for 15 h at 37 ºC
